# Cohort-based strategies as an in-house tool to evaluate and improve phenotyping robustness of LC–MS/MS lipidomics platforms

**DOI:** 10.1007/s00216-024-05404-8

**Published:** 2024-06-28

**Authors:** Benedikt Zöhrer, Cristina Gómez, Joaquim Jaumot, Helena Idborg, Signe S. Torekov, Åsa M. Wheelock, Craig E. Wheelock, Antonio Checa

**Affiliations:** 1 Respiratory Medicine Unit, Department of Medicine Solna, Center for Molecular Medicine, Karolinska Institutet, 171 76 Stockholm, Sweden; 2 Department of Respiratory Medicine and Allergy, Karolinska University Hospital, 171 76 Stockholm, Sweden; 3 Unit of Integrative Metabolomics, Institute of Environmental Medicine, Karolinska Institute, 171 65 Solna, Sweden; 4 Department of Environmental Chemistry, IDAEA-CSIC, Jordi Girona 18-26, E08034 Barcelona, Spain; 5 Division of Rheumatology, Department of Medicine, Karolinska Institutet and Karolinska University Hospital, Solna, Stockholm Sweden; 6 Department of Biomedical Sciences, University of Copenhagen, Copenhagen, Denmark

**Keywords:** Bioanalytical methods, Sphingolipids, Lipidomics, LC-MS/MS

## Abstract

In recent years, instrumental improvements have enabled the spread of mass spectrometry–based lipidomics platforms in biomedical research. In mass spectrometry, the reliability of generated data varies for each compound, contingent on, among other factors, the availability of labeled internal standards. It is challenging to evaluate the data for lipids without specific labeled internal standards, especially when dozens to hundreds of lipids are measured simultaneously. Thus, evaluation of the performance of these platforms at the individual lipid level in interlaboratory studies is generally not feasible in a time-effective manner. Herein, using a focused subset of sphingolipids, we present an in-house validation methodology for individual lipid reliability assessment, tailored to the statistical analysis to be applied. Moreover, this approach enables the evaluation of various methodological aspects, including discerning coelutions sharing identical selected reaction monitoring transitions, pinpointing optimal labeled internal standards and their concentrations, and evaluating different extraction techniques. While the full validation according to analytical guidelines for all lipids included in a lipidomics method is currently not possible, this process shows areas to focus on for subsequent method development iterations as well as the robustness of data generated across diverse methodologies.

**Graphical abstract:**

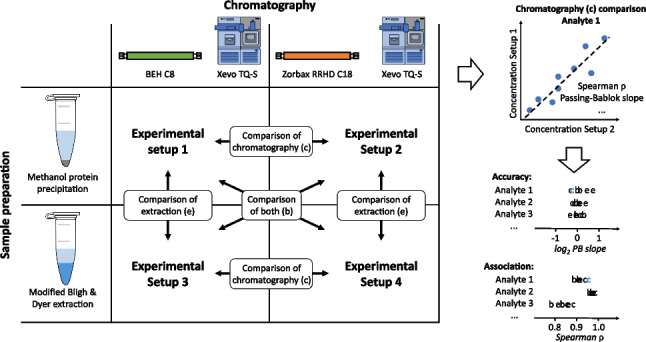

**Supplementary Information:**

The online version contains supplementary material available at 10.1007/s00216-024-05404-8.

## Introduction

The simultaneous analysis of small molecules (< 1200 Da) via mass spectrometry (MS)–based analytical platforms is currently one of the main strategies for cohort phenotyping in biomedical research. This strategy has led to relevant discoveries both for individual metabolites and at the pathway level [1–4].

In cohort phenotyping studies, large inter-individual biological variability, even within groups, often results in the necessity of large amounts of samples to gain new insights from the data. Even with perfectly designed studies, it is hard to reduce or even assess this biological variability in a real-world situation. Therefore, decreasing potential sources of variability in all parts of the analytical pipeline or, at least, assessing potential weak spots of in-house metabolite quantitation platforms becomes critical, even for exploratory studies. These weak spots arise from some pitfalls inherent to the technique that cannot be avoided and thus must not be overlooked [5]. Among these problems, matrix effects can be highlighted, as they not only do occur, but also are hard to evaluate in biological matrices due to their dependence on the individual [6].

The importance of matrix effects in quantification was established in parallel to the widespread adoption of mass spectrometry, and strategies for their assessment have been evaluated ever since [7–11]. Matrix effects are, with different levels of coverage, highlighted in the different guidelines for method validation [12, 13]. One of the main challenges is how to evaluate and tackle the quantification of endogenous metabolites in biological matrices. Thus, strategies that can be useful in drug monitoring scenarios, where non-endogenous compounds are frequently the target metabolites, do not easily translate to primary metabolites as, for instance, a totally deployed plasma or serum matrix for these metabolites does not exist. The strong inter-individual within-matrix variability can also depend on the studies where these methods are applied, like individuals with a certain disease, in the case of biomarker studies.

Accurate quantification of a compound is only consistently possible with the use of a labeled internal standard (LIS), as this is the only known way to reliably compensate for differences in ionization by the coeluting compounds extracted from the matrix [6, 14, 15]. However, full coverage with LIS in multi-compound platforms is unfeasible for real-world large-scale multianalyte analyses. Even when using targeted LC-MS/MS platforms, where confidence in compound identification is high, the lack of LIS due to cost or availability carries the risk of yielding non-accurate, non-reproducible results [13]. Moreover, when several LISs of compounds from the same pathway are used, additional problems may arise due to both cross-contributions in selected reaction monitoring (SRM) windows from other compounds and additive effects of mixed impurities. Suitability of surrogate IS, labeled or not, can be assessed during full method validation, but challenges arise when dozens to hundreds of metabolites have to be evaluated. Additionally, the different backgrounds that can be introduced in different studies cannot, in many instances, be covered within the validation. Interlaboratory studies, comparing multiple samples, are a gold standard approach for these evaluations [16]. However, iterative addition of new compounds to the platform makes it challenging to set up the studies to evaluate the methods in a timely manner.

The importance of the LIS in precision has been recently demonstrated in a thorough large-scale study including 71 endogenous metabolites, all with their matching LIS, using three different source materials [17]. The importance in accuracy, on the other hand, can be challenging to show using just a few pooled materials, as each sample is affected on an individual basis, and sample replicates of pooled materials cannot overcome this problem. The accuracy problem may be a minor issue in many biomedical studies depending on their endpoint. If the objective of the study is to find relative differences between groups, even inaccurate concentration values within groups may be compensated when averaging out all the samples. However, this must be assessed for other endpoints, like associations with certain clinical parameters, integration with other -omics or clustering of individuals for endotyping studies within a disease, as accuracy errors may affect the results both via the generation of false positives and negatives.

The use of cohorts has been shown as a strategy to benchmark lipidomic platforms within a laboratory [18, 19]. These approaches have proven their value to show the consistency of results between platforms analyzing the same metabolites at the multivariate level. However, specific problems for individual metabolites might be overlooked when applying multivariate analysis.

Herein, we show how the in-house use of a cohort-based strategy can help to pinpoint the strong and weak points of LC-MS platforms at the individual compound level. The advantages of using multiple samples instead of a few pooled materials and more than one cohort are highlighted. To this end, we show how we evolved our LC-MS/MS sphingolipid determination platform as an example. Evaluation of the results in a study will highlight potential problems for certain compounds, which can serve as the baseline to improve in the next iteration and must be considered in result evaluation meanwhile.

## Material and methods

### Samples

Plasma samples from three different cohorts were included in the study. All individual identifiers were reshuffled to a random number so that they could not be traced back to an individual, as the only relevant parameter was to include differences in matrix composition based on the disease and sample collection. Cohort 1 (C1) included 90 samples from the Lung Obstruction in Adulthood of Prematurely Born (LUNAPRE) cohort [20]. Cohort 2 (C2) included 48 samples from the Interaction Between Appetite Hormones trial [21]. Cohort 3 (C3) consisted of 40 samples from the Karolinska SLE cohort [22]. A description for each cohort is detailed in Electronic Supplementary Material (ESM) 1.

### Standards

A total of 24 sphingolipids (9 ceramides, 6 sphingomyelins, 4 hexosylceramides, 4 lactosylceramides, and 1 dihydroceramide) were consistently analyzed in all cohorts. Additional standards, when available, were used in the application study. All standards were purchased from Avanti Polar Lipids (Alabaster, AL, USA), Larodan (Solna, Sweden), or Matreya LLC (State College, PA, USA). Details are supplied in Tables S1 and S2 in ESM 2.

### Extraction of sphingolipids

Two different methods were used for extraction. The same samples were simultaneously extracted in parallel with both methods. Together with each batch of extraction, a plasma reference material from the laboratory was extracted as quality control of the extraction (QC_Ext_). The blank of the extraction consisted of deionized water samples with the same extraction protocol. For each batch, samples were thawed at 4 °C and then vortexed for 20 s. Afterwards, a volume of 25 µl of each sample was aliquoted on two Eppendorf tubes (one per extraction procedure for cohorts 1 and 3). Then, 10 µl of the internal standard mixture (different for each study, see Table S3 in ESM 2) was added to each sample. Tubes were then capped, vortexed for 10 s, and allowed to equilibrate with the internal standard for 10 min at room temperature (20 °C). Afterwards, sphingolipids were extracted from samples using one or two different extraction protocols, depending on the cohort. For both methods, to prepare the quality control of injection (QC_inj_), 20 µl of each sample was pooled into a 4-ml glass vial, homogenized, and aliquoted back in individual vials of 80 µl. All extracted samples and QCs were stored at − 20 °C until the day of analysis. The protocol of extraction is summarized in Figure S1 in ESM 3.

#### Methanol protein precipitation extraction

A volume of 250 µl of methanol was added to each sample. Eppendorf tubes were then closed and vortexed for 10 s. Afterwards, samples were sonicated for 15 min on an ice bath (Fisherbrand, Fisher Scientific, UK) to facilitate protein precipitation and avoid temperature increases. Samples were then centrifuged (5430R, Eppendorf, UK) at 10,000 g for 10 min. Two aliquots of 80 µl were finally transferred to two LC-MS vials equipped with a 150-µl insert (Waters).

#### Modified bligh and dyer extraction

First, a volume of 75 µl of deionized water was added to each sample. After a 10-s vortex step, 570 µl of methanol:chloroform (2:1, v/v) was added to each sample. Eppendorf tubes were then closed and vortexed for 10 s. Afterwards, samples were sonicated for 15 min on an ice bath. This was followed by the sequential addition of 190 µl of chloroform and 190 µl of deionized water with a 20-s vortex step after each addition. Samples were then centrifuged at 12,000 g for 10 min. The organic lower layer was carefully recovered and transferred to another Eppendorf tube. To homogenize the recovery, the transferred volume was kept constant at 320 µl per sample. The organic extract was then evaporated using a Turbovap system (Biotage, Sweden) (around 20 min per batch). Afterwards, samples were reconstituted in 275 µl of methanol and vortexed for 10 s. Two 80-µl aliquots were finally transferred to two LC-MS vials equipped with a 150-µl insert.

### UPLC-MS/MS determination of sphingolipids

Each sphingolipid extract was quantified using different UPLC-MS/MS determinations, with chromatography only differing in the column, gradient, and temperature. All injections were performed within a maximum of 5 days after the extraction with extracts stored at − 20 °C until injection. Separation of sphingolipids was performed on an ACQUITY UPLC System from Waters Corporation with a Sample Manager cooled to 8 °C (both from Waters Corporation, Milford, MA, USA). For both methods, mobile phases were kept constant. Mobile phase A consisted of 5 mM ammonium formate/0.2% formic acid in water and mobile phase B consisted of 5 mM ammonium formate/0.2% formic acid in MeOH. A volume of 3 μl was injected for cohort 1 (first experiment) and 2.5 μl was injected for the rest of experiments.

#### Chromatographic method 1

Separation was carried out on a Zorbax Rapid Resolution RRHD C18 Column, 80 Å, 1.8 µm, 2.1 mm × 100 mm (Product Number: 758700-902) equipped with a guard column (Zorbax, 80 Å, 1.8 µm, 2.1 mm × 5 mm, Product Number: 823750-937), both from Agilent (Santa Clara, CA, USA). The UPLC method had a flow rate of 450 μl/min and a column temperature of 40 °C. The chromatographic gradient and the retention times for each compound are detailed in Tables S1, S2, and S4 (ESM 2).

#### Chromatographic method 2

Separation was carried out on an ACQUITY UPLC BEH C8 Column, 130 Å, 1.7 µm, 2.1 mm × 150 mm (Product Number: 186003377) equipped with a guard column (ACQUITY UPLC BEH C8 VanGuard Pre-column, 130 Å, 1.7 µm, 2.1 mm × 5 mm, Product Number: 186003978), both from Waters, as previously detailed [23] with minor modifications to include sphingoid bases in the same analysis. The UPLC method had a flow rate of 375 μl/min and a column temperature of 50 °C. The chromatographic gradient and the retention times for each compound are detailed in Tables S1 and S4 (ESM 2).

#### Chromatographic method 3

Separation was carried out on an ACQUITY Premier CSH C18 FIT Column, 130 Å, 1.7 µm, 2.1 mm × 100 mm with a Vanguard FIT cartridge (Product Number: 186009464), from Waters. The UPLC method had a flow rate of 350 μl/min and a column temperature of 45 °C. The chromatographic gradient and the retention times for each compound are detailed in Tables S2 and S4 (ESM 2).

### Tandem mass spectrometry detection

Sphingolipids were determined using a Waters Xevo® TQ-S system equipped with an electrospray ion source (ESI) and ScanWave™ collision cell technology operating in the positive mode. A class-specific selected reaction monitoring (SRM) transition for each sphingolipid was used. To include all compounds within a single analysis, the SRM transitions were first optimized, with external standards from each sphingolipid, and then, the ones saturating the signal were deoptimized to bring each compound within the range of linearity. Specific SRM transitions, cone voltages, and collision energies for each compound on each cohort are detailed in Tables S1 and S2 (ESM 2). Additionally, the total ion current (TIC) chromatogram between m/z 100 and 1000 was acquired for three samples using the three chromatographic methods (Figure S2 in ESM 3).

### Sequence of analysis and data processing

Samples belonging to each extraction method were injected independently on both chromatographic methods. To minimize biases introduced by the injection of one sample into the next, samples were injected in the same order. One QC_inj_ was injected every 14 samples to monitor instrument performance. To determine the precision of the extractions minimizing any potential instrumental drifts, QC_Ext_ were injected at the end of the sequence in randomized order.

An independent Targetlynx file was created with processed data from each method.

### Data analysis

All statistical analyses were performed using R version 4.3.2. Passing-Bablok was performed using the *mcr* package. Data manipulation and presentation were performed using the *tidyverse* and *DT* packages. All data and code employed in the calculations and figure generation are provided in Supplementary Data 1 (ESM 4) and Supplementary Data 2 (ESM 5).

## Results and discussion

### Rationale, description, and performance of the analytical platforms

Increased availability of external and internal standards, chromatographic, and MS improves over time, as well as the need for increasing throughput drives the constant development of quantitative multianalyte platforms. Proper evaluation of these platforms collides with the time frame of interlaboratory studies. Moreover, it may be extremely challenging to find laboratories covering all metabolites present in the in-house platform. We describe here a workflow for in-house evaluation of multianalyte measuring LC-MS platforms. The use of the herein presented workflow enables retrospective evaluation of the platform, showing strengths and pitfalls of the methodology, which can then be used as the following upgrade in the sequential development and should also be considered during data analysis.

Combinations of two extraction and three chromatographic methods for the targeted determination of sphingolipids were used as a base of the study. These methods have been employed in different studies of the laboratory for the quantification of circulating sphingolipids over the years [2, 23, 24]. Over time, modifications in the method have been introduced to increase throughput, decrease required starting material, and improve separation at the moment a coelution was noticed for the first time. Additionally, LISs were added as they became commercially available.

Evaluation of the analytical parameters for multianalyte methods, especially in the case of endogenous compounds, is challenging. This problem happens even in the case of structurally related compounds, such as sphingolipids. Therefore, minor constant improvements for the methodologies may be delayed or not performed at all in order not to lose track or to avoid inconsistent values with previous results. In the laboratory, we set up a method for the evaluation on how changes introduced in the platform may affect the results in both the short and long term. The platforms were labeled according to the chromatographic and extraction methods as *C18_Met*, *C18_BD*, *C8_Met*, and *C8_BD*, where Met and BD stand for methanol and modified Bligh and Dyer extraction, respectively. The C18 column used in this section was the Zorbax C18 RRHD. Because populations analyzed may affect the obtained results, and to avoid over- and underestimation of the performance based on a single experiment, we used anonymized subsets of samples from cohorts analyzed at the laboratory over time to perform the evaluations. To narrow confounding contributions of other parameters on the interpretation, compared analytes, reconstitution volume, and mobile phases were kept constant for all the platforms and experiments were reported.

First, a full experiment (2 extractions × 2 chromatographic separations) was set using cohort 1 (*n* = 90 samples) to evaluate the suitability of transferring our original *C8_BD* (23) to a *C18_Met* (2) setting. The complementary *C8_Met* and *C18_BD* settings were also evaluated to aid in the interpretation of the results. Then, results from cohort 1 were used to introduce further improvements into the method and select a reasonable number of samples needed for future development evaluations. Based on these criteria, results obtained in cohort 1 were validated in cohort 3 (2 extractions × 2 chromatographic separations) after a partial validation using cohort 2 (only comparing two chromatographic separations). Modifications introduced between cohorts are summarized in Table S5 (ESM 2). These two additional cohorts were selected to study applicability to typical diseases where sphingolipids are determined, like patients with high BMI (cohort 2) and with immune-mediated diseases (cohort 3).

The present comparisons include selected compounds with good recovery for all platforms (e.g., sphingoid bases are measured in the current methanol extraction–based methodology, but do not have a good recovery with chloroform-based extractions). For accuracy evaluation, we only focused on compounds quantified with external calibration curves from the establishment of the platform. These compounds cover five sphingolipid classes and a total of 24 compounds, with concentrations spanning 4.5 orders of magnitude. To include all samples within the linear range for all compounds in one injection, specific collision energies were optimized for each compound on each chromatographic method. Thus, the optimal conditions were used for compounds in low nanomolar concentrations like Cer(d18:1/12:0), while deoptimized collision energies were used for compounds in the micromolar range, like Cer(d18:1/24:0) (Tables S1 and S2 in ESM 2).

### Precision

Results of %CV for the respective QC_inj_ for each cohort are presented in Figure S3 (ESM 3). For multianalyte platforms, this is in many instances the sole quality control performed to check reproducibility of the area, ratio with an internal standard, or the concentration using one sample periodically injected during the study. Out of the 24 quantified sphingolipids, most compounds (cohort 1 = 95%; cohort 2 = 98%; cohort 3 = 96%) presented a %CV_inj_ below 15, a value considered acceptable to perform forward analyses with these types of platforms [25]. As expected, results were consistently better for compounds quantified with their LIS and for the most abundant ones (Figure S3 in ESM 3). For method comparison, this allows attributing potential inconsistencies to other factors when comparing the different platforms. For these runs, this could be a result of the abundances of the compounds in circulation, as no LIS was available for the low abundant ones.

### Accuracy

The importance of accuracy in exploratory biomedical analyses is lower than in other setups (e.g., when a decision needs to be taken depending on the value). However, having an estimation of the actual concentration, or at least its order of magnitude, can provide useful biological insights.

**Fig. 1:**
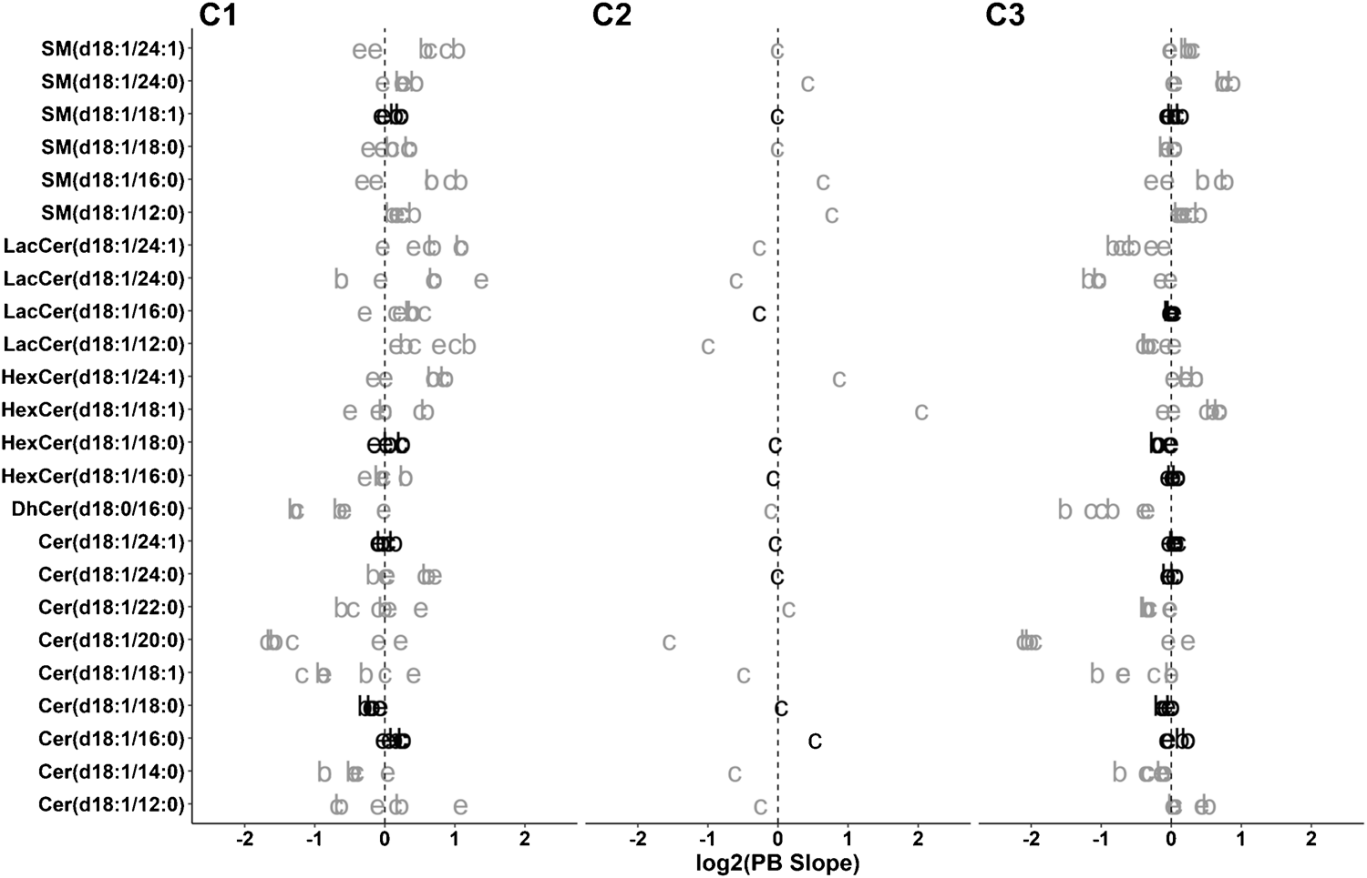
Passing-Bablok regression slopes for all possible method comparisons for C1, C2, and C3. Slopes are depicted as log_2_ so that the deviation of the ideal slope of log_2_ (1) = 0 represents comparable changes in either direction. Letters represent the change between the compared methods (c, column; e, extraction; b, both). For C2, no comparison of extraction was performed. Compounds quantified with their own LIS are presented in black. Dashed vertical line represents the value where calculated concentrations are the same

Agreement on the calculated concentrations’ accuracy between methods was estimated by using Passing-Bablok regression, a linear regression between the concentrations measured by two distinct analytical methods [26]. The concentrations calculated by both methods are considered comparable, when the confidence intervals of the slope and intercept contain 1 and 0, respectively. The slope and intercept of the six possible extraction vs chromatographic method combination pairs were calculated for all compounds within each cohort (Fig. 1, Table S6 in ESM 2). As expected, the use of compound-specific LISs resulted in a more robust agreement between the calculated concentrations (Table S7 in ESM 2).

Table S8 (ESM 2) shows the number of cases where the slope range with confidence intervals contained 1 and the intercept contained 0. As can be seen also in Fig. 1, this number was larger when extraction was modified, relative to changes in both the chromatographic method and both factors simultaneously. This shows that the accuracy was affected more strongly by changes in the chromatography than by the extraction technique in this particular setting. Moreover, the proportion of cases where the intercept contained 0 was larger than the cases where the slope contained 1. This indicates that factors affecting the quantification are proportional to their concentration, which in turn suggests that the accuracy is influenced by the background.

Taking together these results, we conclude that, in this configuration, differences in the background generated by both extraction methods could be globally compensated better than those generated by the chromatographic separation of coelutions. However, this may be heavily dependent on the background recovered during the extraction and it is possible that it cannot be generalized. This could also be related to the impossibility of building proper matrix-matched calibration curves, due to the ubiquitous presence of sphingolipids in circulation. Additionally, samples extracted in this study with the BD method were reconstituted to match the dilution factor of protein precipitation with methanol. Different reconstitution volumes or proportions of the solvents may result in less consistent agreement for the extraction.

The best way to properly ensure consistent calculated concentrations was, as expected [6], the use of LISs (Table S9 in ESM 2). This was shown in cohort 1 and reproduced in cohorts 2 and 3, where more LISs were used (Fig. 1, Table S9 in ESM 2), bringing the slope closer to 1 (log_2_ closer to 0) for their corresponding parent compounds. Thus, accuracy consistency between platforms for Cer(d18:1/24:0), HexCer(d18:1/16:0), and LacCer(d18:1/16:0) was improved (Fig. 1), while the results for the other compounds remained unchanged. Additionally, fold changes in the calculated levels caused by the background for compounds like Cer(d18:1/20:0) were reproduced in C2 and C3, where two different types of backgrounds were tested. Finally, the workflow shows that 40 samples were enough to capture these effects.

### Association

**Fig. 2:**
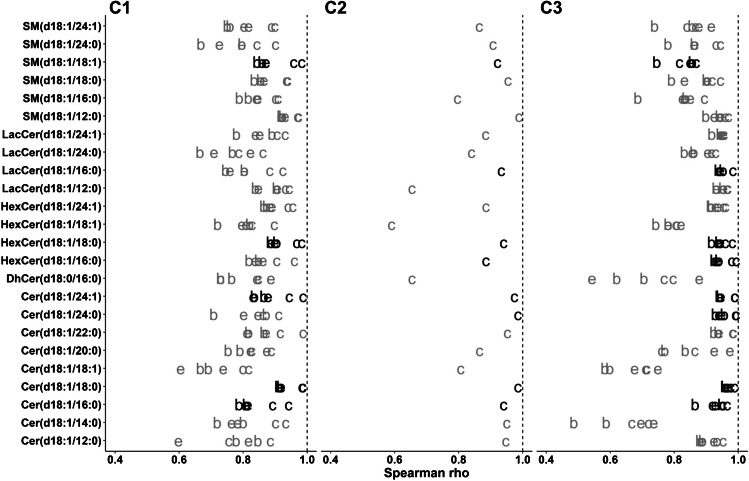
Spearman’s correlation for all possible method comparisons for C1, C2, and C3. Letters represent the change during the analyses (c, column; e, extraction; b, both). For C2, no comparison of extraction was performed. Compounds quantified with their own LIS are presented in black. Dashed vertical line represents the maximum value

Many small-molecule exploratory biomedical analyses aim at establishing associations between the compound of interest and other biomolecules or clinical scores. For these associations, actual quantification is less relevant and, many times, associations using compound areas or ratios with a class-specific internal standard suffice. Simultaneous comparisons of different methods enable the identification of the base error that we should expect when performing an association with another parameter measured with a different degree of precision. For a rapid inspection of these associations, all possible calculations (2 extractions and 2 chromatographic separations in cohorts 1 and 3, and 1 extraction and 2 chromatographic separations in cohort 2) can be performed as before and presented in a single graph. In order to avoid problems with extreme outliers, we used Spearman’s correlation (Fig. 2). The same representation was obtained using Pearson’s correlation instead, showing no large differences for the samples analyzed in the current study (Figure S4 in ESM 3).

Again, the use of the own LIS resulted in more consistent results regarding comparisons for cohort 1 (Fig. 2, Figure S4 in ESM 3). However, in this case, more consistent results were obtained when changing the column, relative to the change of the extraction or both. A potential explanation is that the same extract was injected on both columns, which introduced lower variability than the process of extraction.

Parallel to the addition of more LISs, the concentration of already used LISs was modified for cohorts 2 and 3 based on interim optimizations (Table S3 in ESM 2). As a result, consistency of the comparisons improved not only for the three compounds now determined with their LISs, but also for all the compounds that already had their own LIS, with the exception of SM(d18:1/18:1) (Fig. 2, Figure S4 in ESM 3).

On the other hand, results for compounds without their own LIS were not always improved, and *ρ* values were even lower for compounds like Cer(d18:1/14:0) and DhCer(d18:0/16:0) in cohort 3. Results for DhCer(d18:0/16:0) can be attributed to the lack of resolution of the compound from an endogenous peak on the C_8_ column, one of the motivations for changing the chromatographic method (Figure S5, ESM 3). The close coeluting peak shown in Figure S5 (ESM 3) was not apparent in previous cohorts analyzed in the lab, as only resulted in some tailing for some BD extractions. The peak became apparent when using methanol for the extraction and using C18 for the separation. Importantly, the changes in relative abundances of DhCer(d18:0/16:0) and the interference in different samples would have been observed when comparing the methods even with full coelution for the C8 method.

### Quantile analyses

Another classical type of analysis with metabolite data is to categorize samples into quantiles according to their content of the compounds [1, 2, 27]. Kaplan-Meier estimates, conditional logistical regression analysis, or multivariable Cox proportional hazard models are then calculated based on these quantiles. Again, we can evaluate the consistency of these quantile generations depending on the method and the IS used. Based on the low number of samples, the tertiles for each compound determined using each method were computed for the six possible comparisons. We established a tertile scoring system with a positive score for compounds with tertile matching, and a null or negative score for compounds with one or two tertiles of difference, respectively (Figure S6 in ESM 3).

Individually, this is not a very informative measurement, as the benchmark of what represents a good value will depend on the range and distribution of each compound in a given cohort. If many compounds have values similar to the limits of the tertiles, the scoring system could be lower regardless of the method reproducibility. However, the simultaneous representation of all possible combinations enables the evaluation of potential limitations of these quantile analyses for certain compounds.

**Fig. 3:**
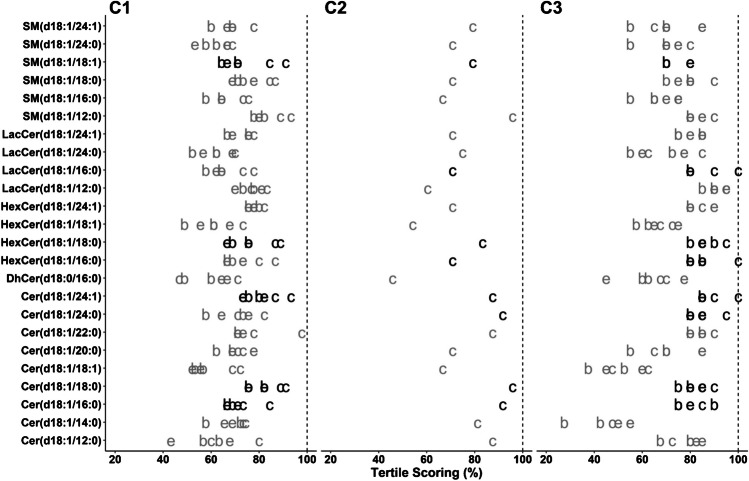
Tertile scoring for all possible method comparisons for C1, C2, and C3. Letters represent the change during the analyses (c, column; e, extraction; b, both). For C2, no comparison of extraction was performed. Compounds quantified with their own LIS are presented in black. Dashed vertical line represents the maximum value

Results for the tertile scoring of cohort 1 show that the inclusion of LISs did not result in an obvious improvement as observed in other calculations, as values for compounds with their own LIS were not significantly closer to a 100% score than the ones without (Fig. 3). This was also observed for the three additional compounds in cohort 2 determined with their LIS relative to cohort 1. The optimization in LIS concentration obtained in cohort 3 resulted in improved scoring for all compounds relative to cohort 1, with the exception of SM(d18:1/18:1). As observed in the Spearman correlation (Fig. 2), performance evaluation for Cer(d18:1/14:0) was lower for these analyses, highlighting the complexity of improving the performance of the method for all compounds.

### Class LIS selected sub-analyses

**Fig. 4:**
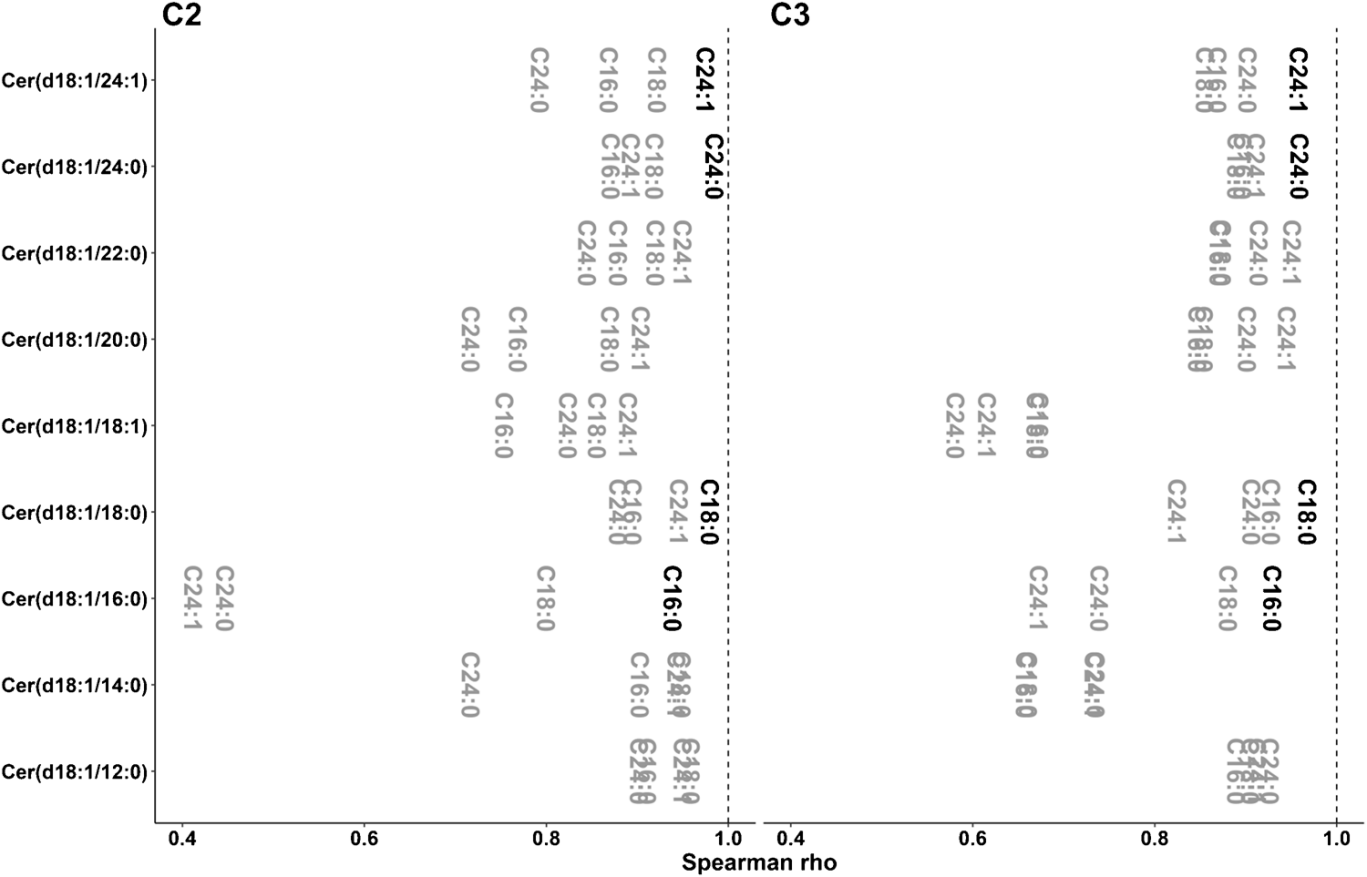
Spearman’s correlation for all possible combinations of ceramide/LIS for C2 and C3. Text represents the acyl chain of the d_7_-labeled internal standard used for the quantification. For C3, results are presented as the average value per compound/LIS obtained for all six possible comparisons (2 extractions × 2 chromatographic separations). For individual values, see Table S10 (ESM 2). Values calculated using the own LIS was used are presented in black. Dashed vertical line represents the maximum value

**Fig. 5:**
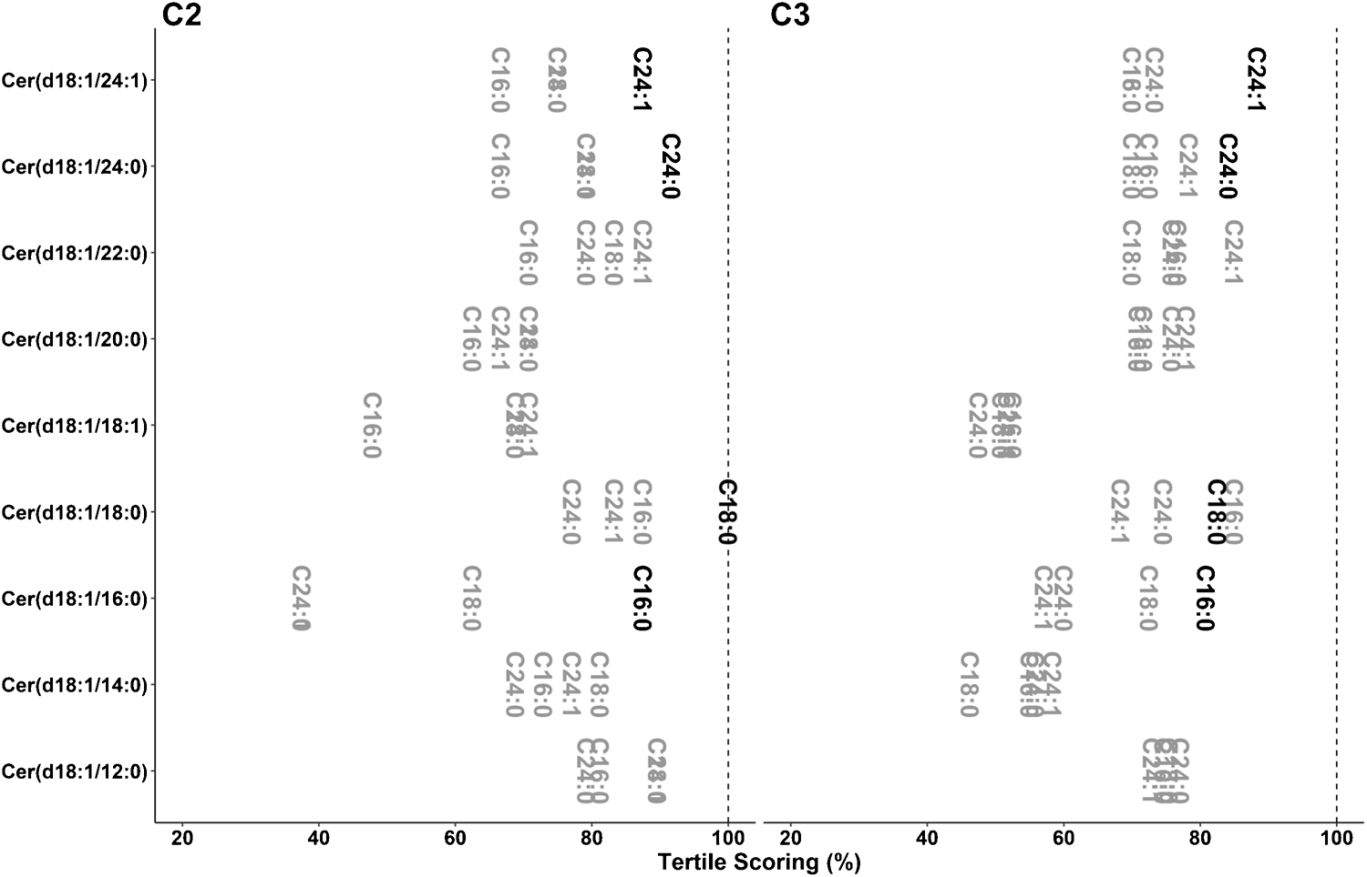
Tertile scoring for all possible method comparisons for C2 and C3. Text represents the acyl chain of the d_7_-LIS used for the quantification. Results are presented as the average value per compound/LIS obtained for all the injected methods (*n* = 2 and 4, respectively). For individual values, see Table S10 (ESM 2). Results where the own LIS was used are presented in black. Dashed vertical line represents the maximum value

The previous analyses show the effects of using the own LIS on circulating sphingolipid quantification for three different cohorts. Those compounds without their own LIS were quantified using a surrogate LIS (with the exception of LacCers in cohort 1) from the same class for the quantification, a common approach. Previous studies have shown that when the own LIS is not available, the use of quality controls from the same matrix enables the selection of the best surrogate IS [6]. This approach is generally applicable but cannot cover all potential background possibilities that will happen at the individual level (Figure S2 in ESM 3). We thus used the ceramide data from cohorts 2 and 3, where four different ceramide LISs, namely Cer(d18:1/16:0)-d_7_, Cer(d18:1/18:0)-d_7_, Cer(d18:1/24:1)-d_7_, and Cer(d18:1/24:0)-d_7_, eluting in four chromatographic areas were employed. We evaluated all possible combinations *ceramide/labeled ceramide IS*. Then, we performed the same comparisons as for the whole sphingolipid panel to simultaneously evaluate both the advantage of the own LIS and the robustness of a surrogate LIS in different configurations. Results are summarized in Figs. 4 and 5 as well as Figures S7 and S8 (ESM 3). For cohort 3, for each analyte/LIS, the average for all six comparisons (between 2 chromatographies and 2 extractions) is displayed. Results for individual method comparisons are reported in Tables S10 to S13 (ESM 2).

Again, the use of the own LIS was the best option in all cases for compounds where it was available (Figure S7 in ESM 3). However, the use of any other ceramide LIS still resulted in acceptable %CV considering a typical 20% threshold for exploratory studies. Additionally, thresholds of 10 and 15% would have resulted in non-acceptable results for only 8.3% and 2.8% of the cases, respectively. In the case of accuracy, results confirmed that it is complicated to achieve consistent values over time unless the own LIS is used (Figure S8, ESM 3). As already observed in Fig. 1, accuracy for Cer(d18:1/16:0) was off for cohort 2. This ceramide also showed the larger difference in Spearman’s *ρ* between the use of its own LIS and any other LIS (Fig. 4). Spearman’s *ρ* values for Cer(d18:1/16:0) are lower when other acyl chains are used as LIS when comparing results in cohorts 2 and 3 (Fig. 4). This shows that when a sole pooled reference material is used for the selection of the most suitable LIS, both over- and underestimated results relative to others may be obtained depending on the actual pooled sample. Additionally, the use of different cohorts allows including potential biases introduced by the specific matrices. Similar conclusions can be extracted from the tertile scoring evaluation (Fig. 5).

### Application of the strategy

The comparison strategy was applied in the next chromatographic development. We switched to the CSH C18 column based on preliminary tests showing better performance for some sphingolipids, especially sphingoid bases. This resulted in a modified TIC background (Figure S2 in ESM 3) and the improved separation of a low abundant compound with the same SRM commonly observed in the early eluting ceramides (Figure S9 in ESM 3). Both tested methodologies using either Zorbax C18 or CSH C18 included 88 consistently detected sphingolipids and 12 internal standards (Table S2 in ESM 2), which were included in the comparison.

We used cohort 1 for the comparison. Based on the previous results, we did not perform the absolute quantification, and thus, the Passing-Bablok was not performed. Results for the %CV, associations, and tertile scoring are presented in Fig. 6, Figures S10–S18 (ESM 3), and Tables S14–S19 (ESM 2).

**Fig. 6:**
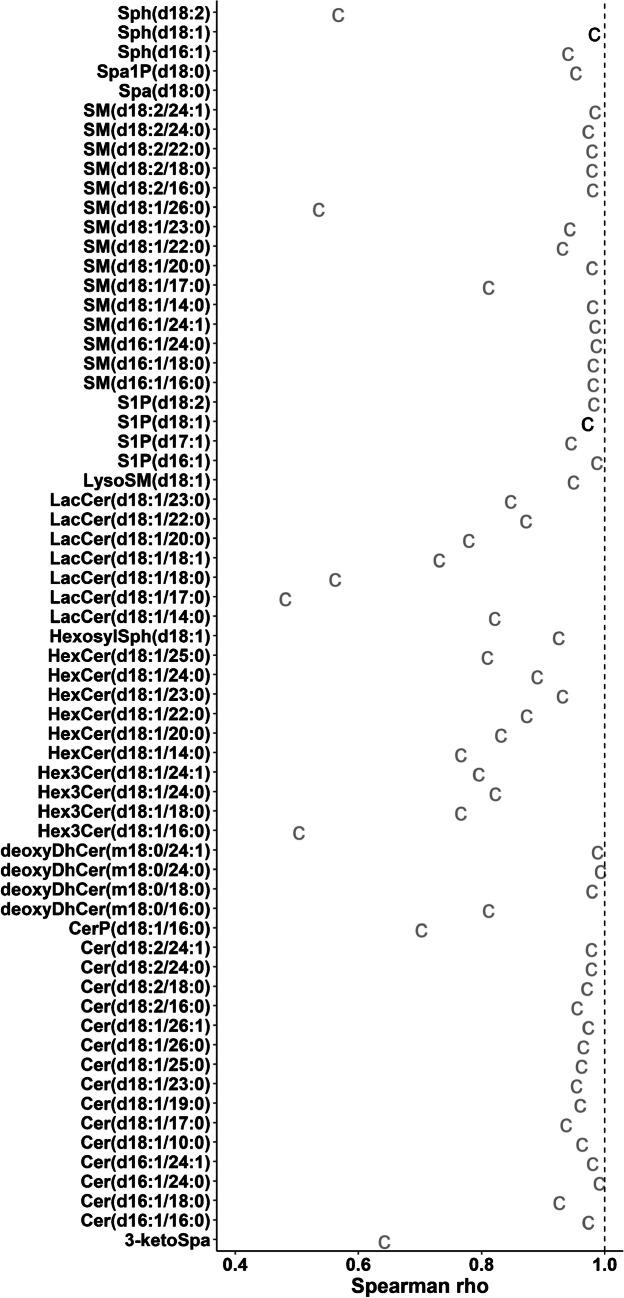
Spearman’s correlation for the comparison between Zorbax C18 and CSH C18 LC-MS/MS methods after the application of the workflow. Only compounds included in both methodologies are shown. Compounds which are quantified on their own labeled internal standard are depicted in black

First, we could corroborate that %CV were lower for sphingoid bases. Specifically, 3-ketosphinganine reproducibility improved with the new column because of an improved chromatographic baseline (Figure S11 in ESM 3). The examination of the associations between Zorbax C18 and CSH C18 injections showed consistent results for most of the sphingolipids, especially those included in the first three experiments (Fig. 6). Inconsistencies in associations for the sphingoid bases were also observed, especially for Spa(d18:0). This compound is an example of how a %CV < 20 could still produce highly irreproducible results, represented by a Spearman *ρ* < 0.3 (Table S14 in ESM2).

Conversely, even though deoxyDhCer(m18:0/16:0) presented better %CV in the Zorbax C18 column, this could be attributed to a coelution that was partially separated using the CSH C18 column. Thus, the worse performance in association and tertile scoring for this compound relative to the other deoxyDhCer were caused by the lack of separation on the Zorbax C18, rather than the lack of reproducibility.

### Final considerations

The need to use the own LIS for the best results in concentration accuracy and in LC-MS-based multianalyte platforms is not controversial. We here show that, as expected, accurate quantification can only be consistently achieved when these LISs are employed. This situation even applies in an experimental configuration where mobile phase composition, operator, and instrumentation are constant. The results also show that consistent concentration values can be obtained when comparing two platforms over time, without these values being necessarily accurate (see Cer(d18:1/20:0), Fig. 1). In general, for compounds without LIS, quantification obtained with these platforms can provide approximate information of the concentration levels for the reported compounds (e.g., high nM), which is informative from a biological standpoint. However, it must always be kept in mind that consistent accuracy (i.e., quantifying the same levels in a reference material across different studies) is not necessarily equivalent to acceptable accuracy (i.e., quantifying the actual value of the compound in the sample).

The data also shows that, regardless of accuracy, values generated between methods are generally consistent for other associations. For example, more than 80% of all the associations had a *ρ* > 0.8 (Table S6 in ESM 2). While modifications included in the method, especially regarding the internal standard concentration, did result in better associations in C3 relative to C1 for compounds with their own LIS, no systematic improvement was observed for the rest of compounds. Similar conclusions can be extracted from the observations regarding the tertile scoring. In general, this shows that results of an optimized platform can be used for further interpretation with confidence, but caution should be taken for compounds that do not present high scores in the internal validation.

Data from the ceramide LIS sub-analyses showed that the use of one class-specific LIS may not be enough to cover all species from that class, especially for challenging matrices like the one in C2. Overall, this approach can be used to select the most suitable LIS surrogate when more than one LIS is available. Based on the results for Fig. 6, for instance, we have incorporated SM(d18:1/26:0)-d_4_ as an internal standard to later eluting sphingomyelins. This also shows that, when no LIS is available, the closest eluting compound is not always the best option, like it can be observed for Cer(d18:1/12:0) and Cer(d18:1/14:0) (Figs. 4 and 5). Importantly, the ideal internal standard assessed in one study may not reproduce in the next. As expected, the optimal scenario occurs, when the compound coelutes, at least partially, with the LIS, as seen with Cer(d18:1/22:0).

Overall, we here show a strategy to internally evaluate the consistency between lipidomic platforms at the individual metabolite level when changes are implemented. This strategy is applicable to the coverage extension of these platforms. With current instrumentation and sample volume requirements, comparisons between two platforms can be easily performed intralaboratory with a subset of study samples in a timely manner. We show that this can be performed with as low as 40 samples, which is a modest amount considering that these methods are developed for their application to large cohorts [2]. This strategy is not possible if one of the platforms cannot cover certain lipid species, like S1P with chloroform-based extractions or if there is a coelution. In those circumstances however, choosing the best methodology is trivial.

Finally, the strategy also enables the inspection of sources of inconsistency, like the presence of total or partial coelutions. It also shows the importance of these inconsistency sources on basic statistical analysis, even on when the same extract is analyzed at the same laboratory with minor changes between methodologies.

## Conclusions

The generalization of multianalyte platforms in biomedical studies allows the generation of large datasets. This data will have different levels of reliability on a compound-specific basis based on the availability of LIS. The use of in-house strategies allows the internal evaluation of the specific reliability for each metabolite according to the statistical test that will be used. Additionally, in-house strategies can be used to improve different aspects of the methodology, such as identification of coelutions of different compounds with the same SRM, selection of the most appropriate LIS, and the concentrations at which they are added for compounds for which it is not available and comparison of extraction methodologies. While no single methodology is expected to be perfect when targeting dozens to hundreds of compounds, changes in sequential iterations can be in-house evaluated in a timely manner to highlight both inconsistencies to improve in further versions or robustness in the data when using different methodologies.

## Supplementary Information

Below is the link to the electronic supplementary material.

Supplementary file1 (DOCX 32 KB)

Supplementary file2 (XLSX 135 KB)

Supplementary file3 (PPTX 7456 KB)

Supplementary file4 (ZIP 87.6 KB)

Supplementary file5 (ZIP 105 KB)
