# Supplementary material for: Cohort-based strategies as an in-house tool to evaluate and improve phenotyping robustness of LC–MS/MS lipidomics platforms
Source: Anal Bioanal Chem. 2024 Jun 28;416(25):5485–96. doi: 10.1007/s00216-024-05404-8 (PMC11427549; doi:10.1007/s00216-024-05404-8)
Supplement: Supplementary file 1 — Supplementary file1 (DOCX 32 KB) [file 216_2024_5404_MOESM1_ESM.docx]

**Electronic Supplementary Materials 1**

**-**

**Description of the cohorts**

**Cohort-based strategies as an in-house tool to evaluate and improve phenotyping robustness of LC-MS/MS lipidomics platforms**

**Benedikt Zöhrer^1,2^, Cristina Gómez^3^, Joaquim Jaumot^4^, Helena Idborg^5^, Signe S. Torekov^6^, Åsa M. Wheelock^1,2^, Craig E. Wheelock^2,3^, Antonio Checa^3^**

^1^ Respiratory Medicine Unit, Department of Medicine Solna, Center for Molecular Medicine, Karolinska Institutet, 171 76 Stockholm, Sweden

^2^ Department of Respiratory Medicine and Allergy, Karolinska University Hospital, 171 76 Stockholm, Sweden

^3^ Unit of Integrative Metabolomics, Institute of Environmental Medicine, Karolinska Institute, 17165, Solna, Sweden

^4^ Department of Environmental Chemistry, IDAEA-CSIC, Jordi Girona 18-26, E08034 Barcelona, Spain

^5^ Division of Rheumatology, Department of Medicine, Karolinska Institutet and Karolinska University Hospital, Solna, Stockholm, Sweden.

^6^ Department of Biomedical Sciences, University of Copenhagen, Copenhagen, Denmark

**Cohort 1 (C1)**

EDTA-plasma from a total of 90 young adults (18-23 years old) from the Lung Obstruction in Adulthood of Prematurely Born (LUNAPRE) cohort was used (www.clinicaltrials.gov/study/NCT02923648). Individuals were recruited between March 2013 and December 2017 at the Department of Medicine Solna, Karolinska Institutet, Department of Respiratory Medicine and Allergy, Karolinska University Hospital Solna, Stockholm, Sweden. Details of the cohort can be obtained elsewhere (1) The study was approved by the Regional Ethical Review Board in Stockholm, Sweden, case no: 2012/1872–31/4. All participants provided their informed written consent.

**Cohort 2 (C2)**

A total of 48 EDTA-plasma samples belonging to 16 individuals (3 time-points per individual, collected at 0, 8 and 52 weeks) from the Interaction Between Appetite Hormones trial were included (2). The study was approved by the ethical committee in Copenhagen (reference number: H-4-2010-134) and was performed in accordance with the Helsinki Declaration II and with ICH-GCP practice. Participation in the investigation was voluntary and the individuals could at any time retract their consent to participate. A selected number of anonymized samples based on the established criteria obtained from Cohort 1 was reanalyzed from a previous study (3).

**Cohort 3 (C3)**

A total of 40 plasma samples from the Karolinska SLE cohort were used (4). These samples were an anonymized subset of a previously analyzed systemic lupus erythematous cross-sectional study, selected based on the established criteria obtained from Cohort 1. The study was approved by the Stockholm regional Ethics Committee and conducted according to the Declaration of Helsinki’s principles.

**References**

1. Um-Bergstrom P, Hallberg J, Pourbazargan M, Berggren-Brostrom E, Ferrara G, Eriksson MJ, et al. Pulmonary outcomes in adults with a history of Bronchopulmonary Dysplasia differ from patients with asthma. Respir Res. 2019;20(1):102.

2. Iepsen EW, Lundgren J, Dirksen C, Jensen JE, Pedersen O, Hansen T, et al. Treatment with a GLP-1 receptor agonist diminishes the decrease in free plasma leptin during maintenance of weight loss. Int J Obes (Lond). 2015;39(5):834-41.

3. Akawi N, Checa A, Antonopoulos AS, Akoumianakis I, Daskalaki E, Kotanidis CP, et al. Fat-Secreted Ceramides Regulate Vascular Redox State and Influence Outcomes in Patients With Cardiovascular Disease. J Am Coll Cardiol. 2021;77(20):2494-513.

4. Checa A, Idborg H, Zandian A, Sar DG, Surowiec I, Trygg J, et al. Dysregulations in circulating sphingolipids associate with disease activity indices in female patients with systemic lupus erythematosus: a cross-sectional study. Lupus. 2017;26(10):1023-33.
