# Supplementary material for: Cohort-based strategies as an in-house tool to evaluate and improve phenotyping robustness of LC–MS/MS lipidomics platforms
Source: Anal Bioanal Chem. 2024 Jun 28;416(25):5485–96. doi: 10.1007/s00216-024-05404-8 (PMC11427549; doi:10.1007/s00216-024-05404-8)
Supplement: Supplementary file 3 — Supplementary file3 (PPTX 7456 KB) [file 216_2024_5404_MOESM3_ESM.pptx]

## Slide 1
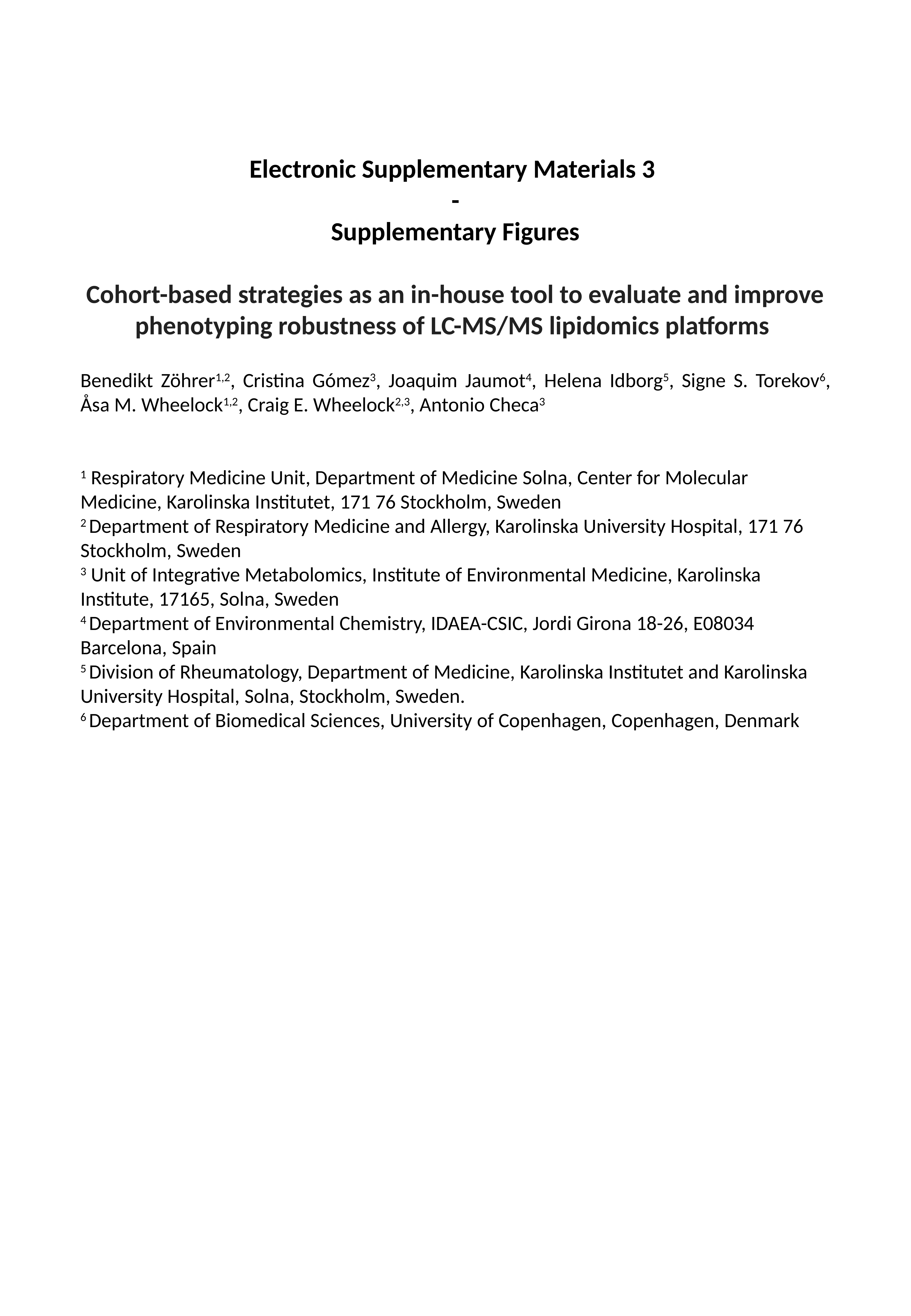

Electronic Supplementary Materials 3
-
Supplementary Figures
Cohort-based strategies as an in-house tool to evaluate and improve phenotyping robustness of LC-MS/MS lipidomics platforms
Benedikt Zöhrer1,2, Cristina Gómez3, Joaquim Jaumot4, Helena Idborg5, Signe S. Torekov6, Åsa M. Wheelock1,2, Craig E. Wheelock2,3, Antonio Checa3
1 Respiratory Medicine Unit, Department of Medicine Solna, Center for Molecular Medicine, Karolinska Institutet, 171 76 Stockholm, Sweden
2 Department of Respiratory Medicine and Allergy, Karolinska University Hospital, 171 76 Stockholm, Sweden
3 Unit of Integrative Metabolomics, Institute of Environmental Medicine, Karolinska Institute, 17165, Solna, Sweden
4 Department of Environmental Chemistry, IDAEA-CSIC, Jordi Girona 18-26, E08034 Barcelona, Spain
5 Division of Rheumatology, Department of Medicine, Karolinska Institutet and Karolinska University Hospital, Solna, Stockholm, Sweden.
6 Department of Biomedical Sciences, University of Copenhagen, Copenhagen, Denmark

## Slide 2
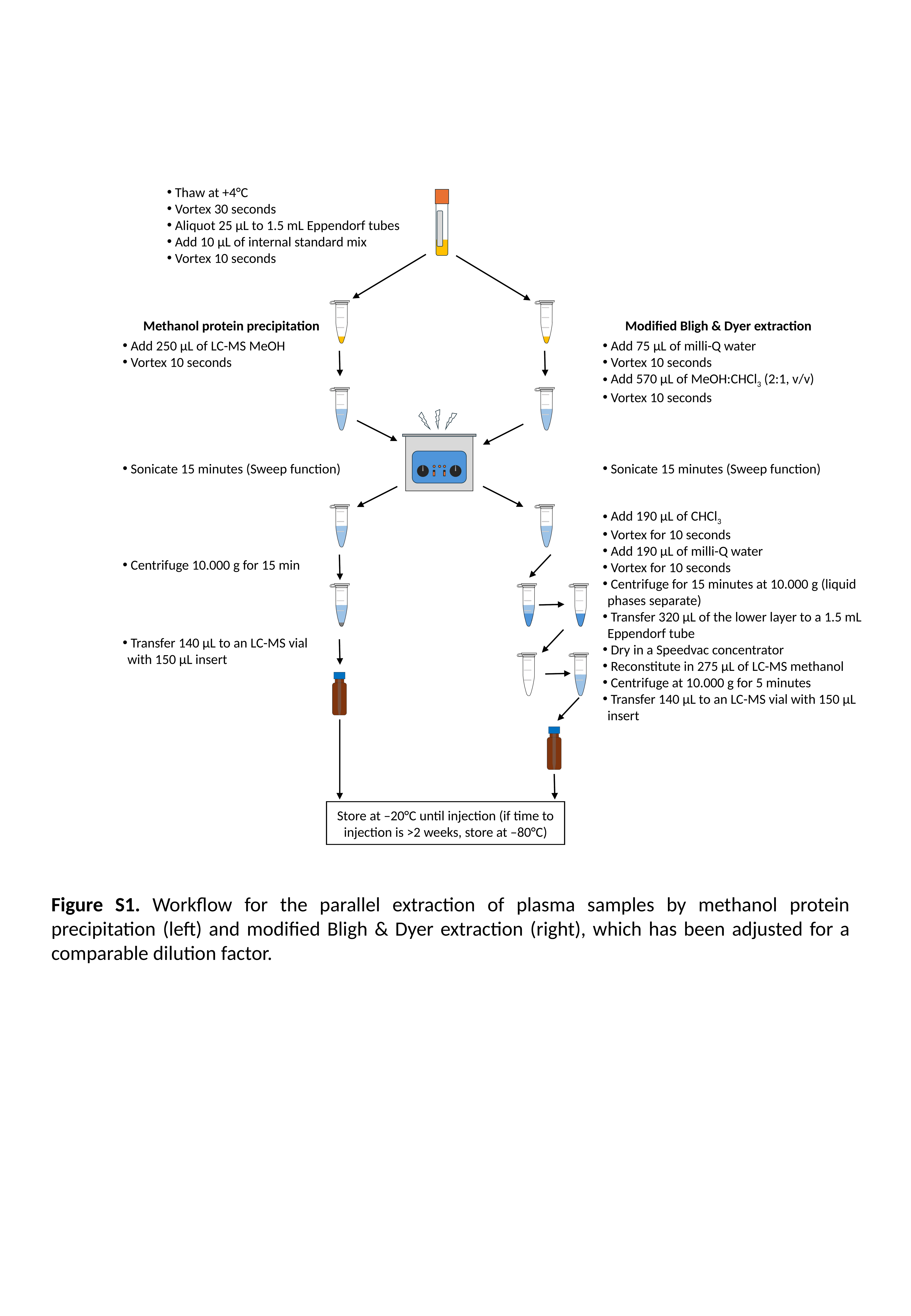

Thaw at +4°C
 Vortex 30 seconds
 Aliquot 25 µL to 1.5 mL Eppendorf tubes
 Add 10 µL of internal standard mix
 Vortex 10 seconds
Methanol protein precipitation
Modified Bligh & Dyer extraction
 Add 75 µL of milli-Q water
 Vortex 10 seconds
 Add 570 µL of MeOH:CHCl3 (2:1, v/v)
 Vortex 10 seconds
 Add 250 µL of LC-MS MeOH
 Vortex 10 seconds
 Sonicate 15 minutes (Sweep function)
 Sonicate 15 minutes (Sweep function)
 Add 190 µL of CHCl3
 Vortex for 10 seconds
 Add 190 µL of milli-Q water
 Vortex for 10 seconds
 Centrifuge for 15 minutes at 10.000 g (liquid phases separate)
 Transfer 320 µL of the lower layer to a 1.5 mL Eppendorf tube
 Dry in a Speedvac concentrator
 Reconstitute in 275 µL of LC-MS methanol
 Centrifuge at 10.000 g for 5 minutes
 Transfer 140 µL to an LC-MS vial with 150 µL insert
 Centrifuge 10.000 g for 15 min
 Transfer 140 µL to an LC-MS vial with 150 µL insert
Store at –20°C until injection (if time to injection is >2 weeks, store at –80°C)
Figure S1. Workflow for the parallel extraction of plasma samples by methanol protein precipitation (left) and modified Bligh & Dyer extraction (right), which has been adjusted for a comparable dilution factor.

## Slide 3
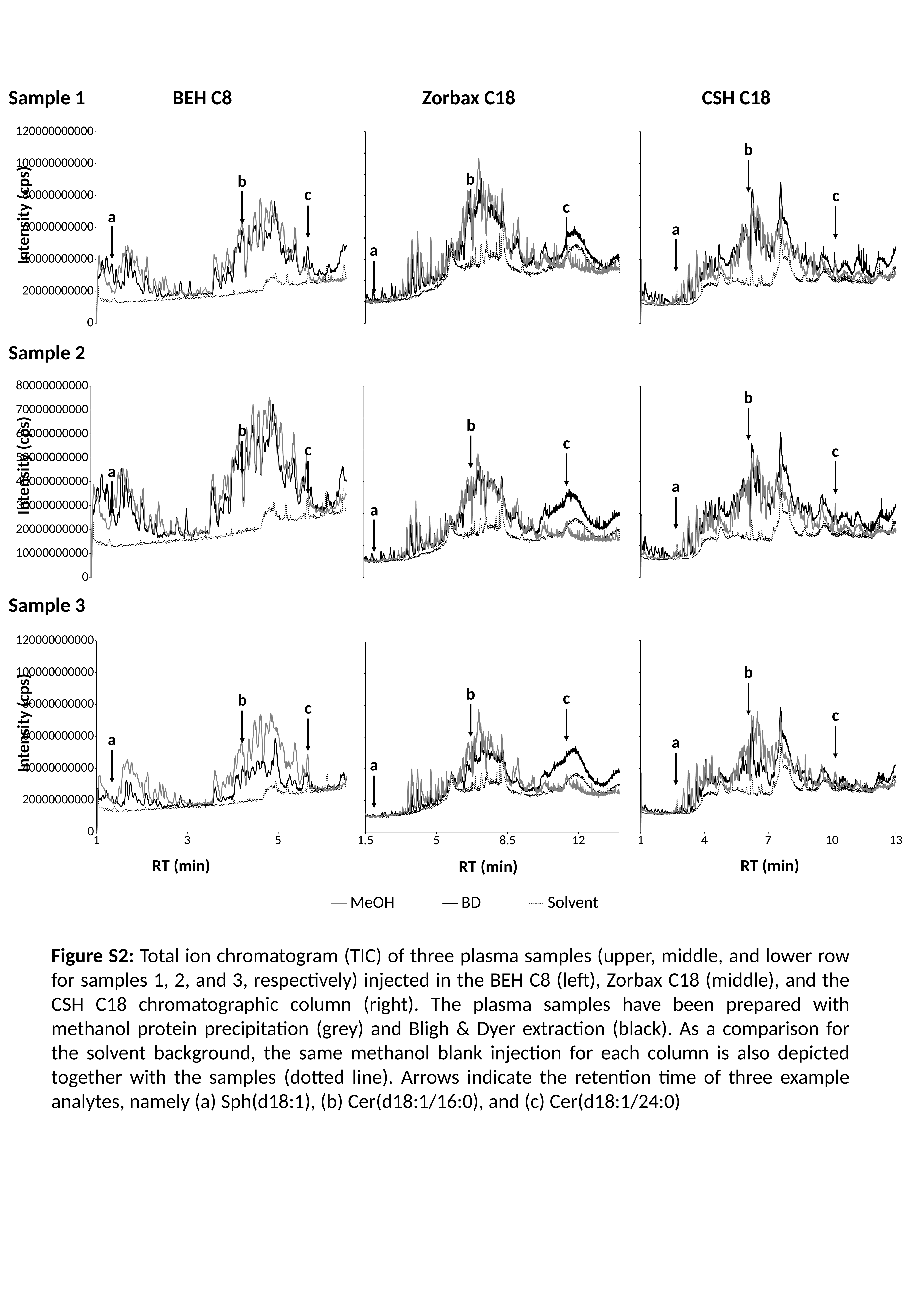

### Chart
| Category | MeOH | BD | Solvent |
|---|---|---|---|
### Chart
| Category | MeOH | BD | Solvent |
|---|---|---|---|
### Chart
| Category | MeOH | BD | Solvent |
|---|---|---|---|Sample 1
BEH C8
Zorbax C18
CSH C18
b
b
b
c
c
c
Intensity (cps)
a
a
a
### Chart
| Category | MeOH | BD | Solvent |
|---|---|---|---|
### Chart
| Category | MeOH | BD | Solvent |
|---|---|---|---|
### Chart
| Category | MeOH | BD | Solvent |
|---|---|---|---|Sample 2
b
b
b
c
c
c
Intensity (cps)
a
a
a
### Chart
| Category | MeOH | BD | Solvent |
|---|---|---|---|
### Chart
| Category | MeOH | BD | Solvent |
|---|---|---|---|
### Chart
| Category | MeOH | BD | |
|---|---|---|---|Sample 3
b
b
c
b
c
c
Intensity (cps)
a
a
a
Figure S2: Total ion chromatogram (TIC) of three plasma samples (upper, middle, and lower row for samples 1, 2, and 3, respectively) injected in the BEH C8 (left), Zorbax C18 (middle), and the CSH C18 chromatographic column (right). The plasma samples have been prepared with methanol protein precipitation (grey) and Bligh & Dyer extraction (black). As a comparison for the solvent background, the same methanol blank injection for each column is also depicted together with the samples (dotted line). Arrows indicate the retention time of three example analytes, namely (a) Sph(d18:1), (b) Cer(d18:1/16:0), and (c) Cer(d18:1/24:0)

## Slide 4
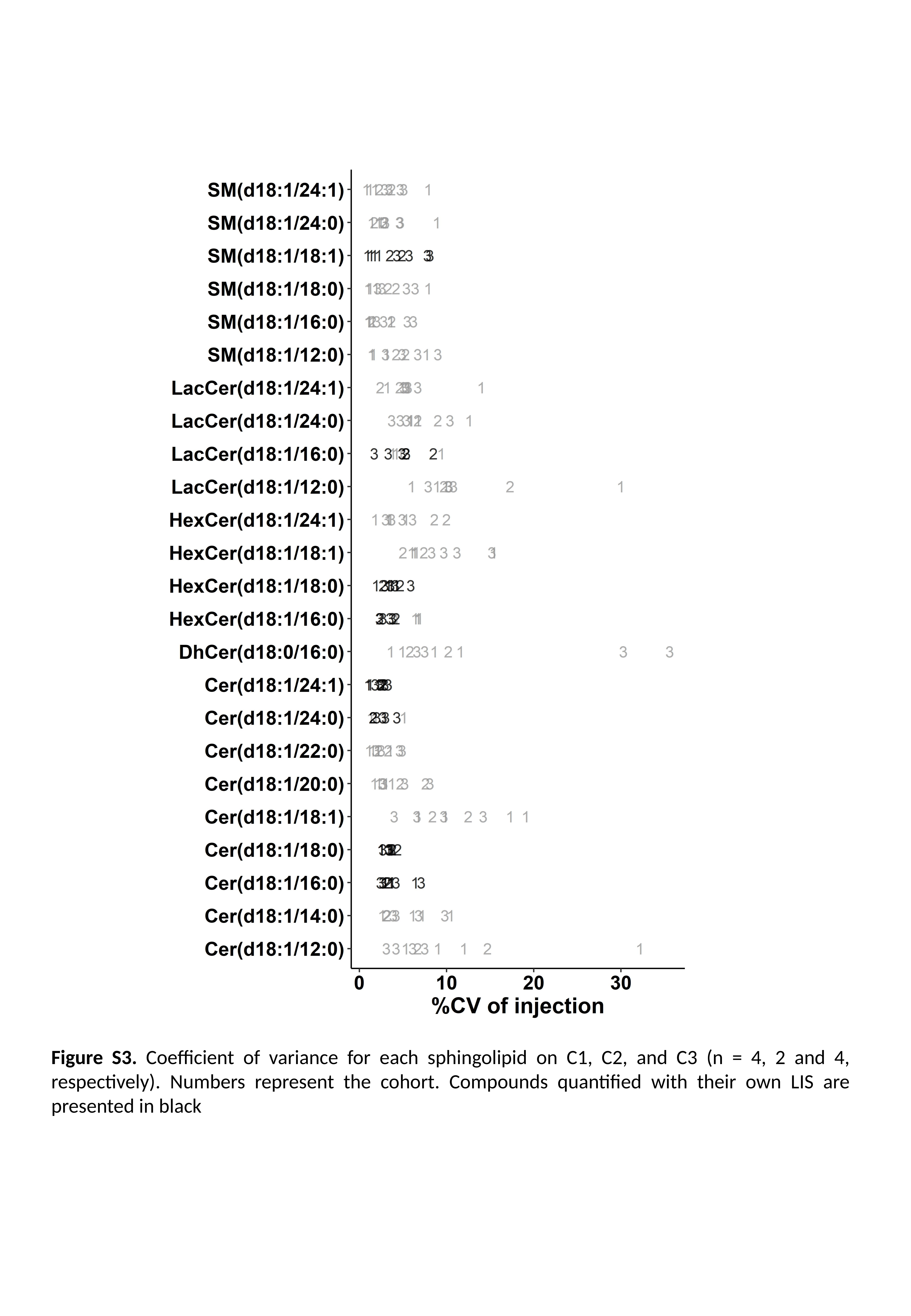

Figure S3. Coefficient of variance for each sphingolipid on C1, C2, and C3 (n = 4, 2 and 4, respectively). Numbers represent the cohort. Compounds quantified with their own LIS are presented in black

## Slide 5
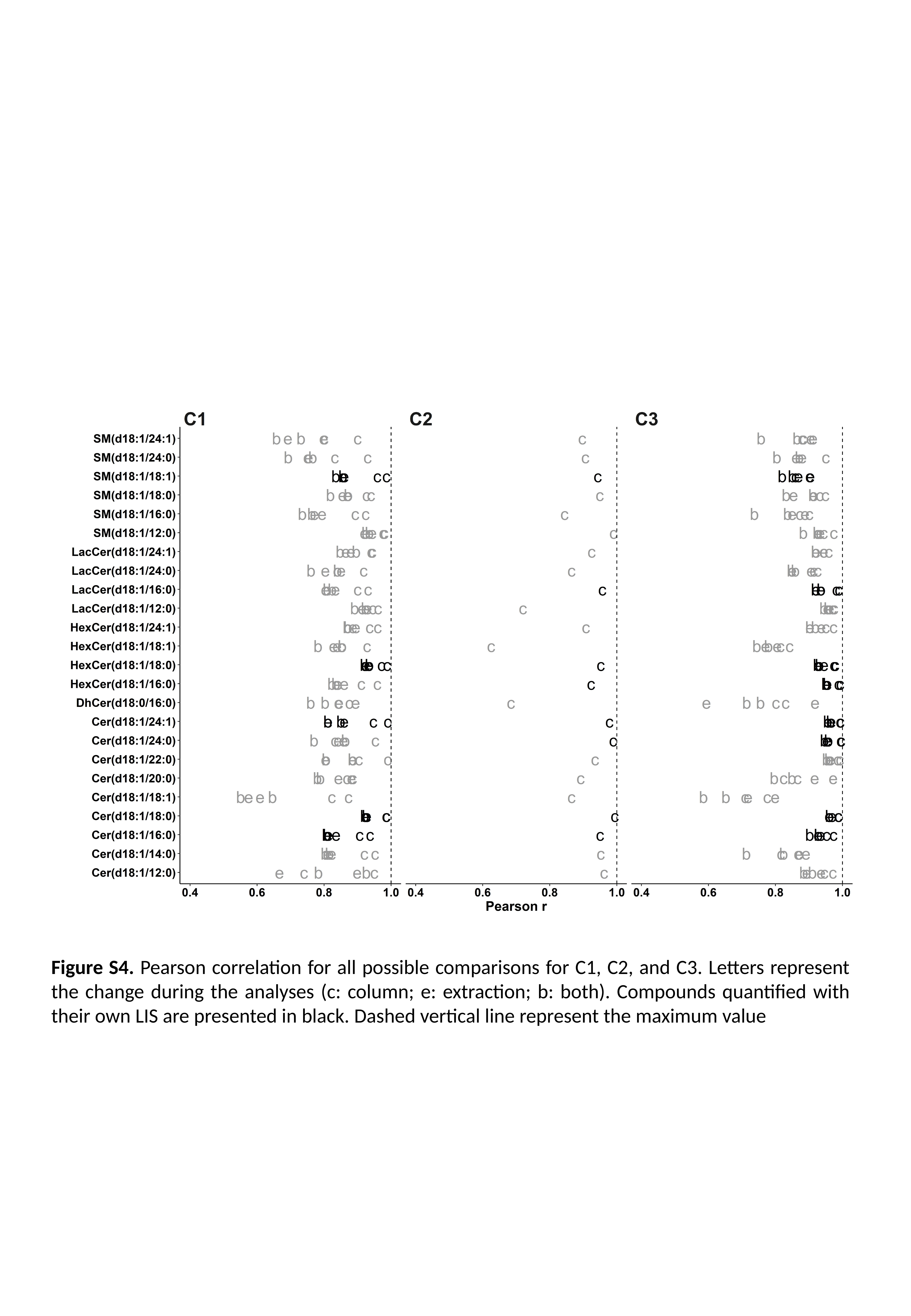

Figure S4. Pearson correlation for all possible comparisons for C1, C2, and C3. Letters represent the change during the analyses (c: column; e: extraction; b: both). Compounds quantified with their own LIS are presented in black. Dashed vertical line represent the maximum value

## Slide 6
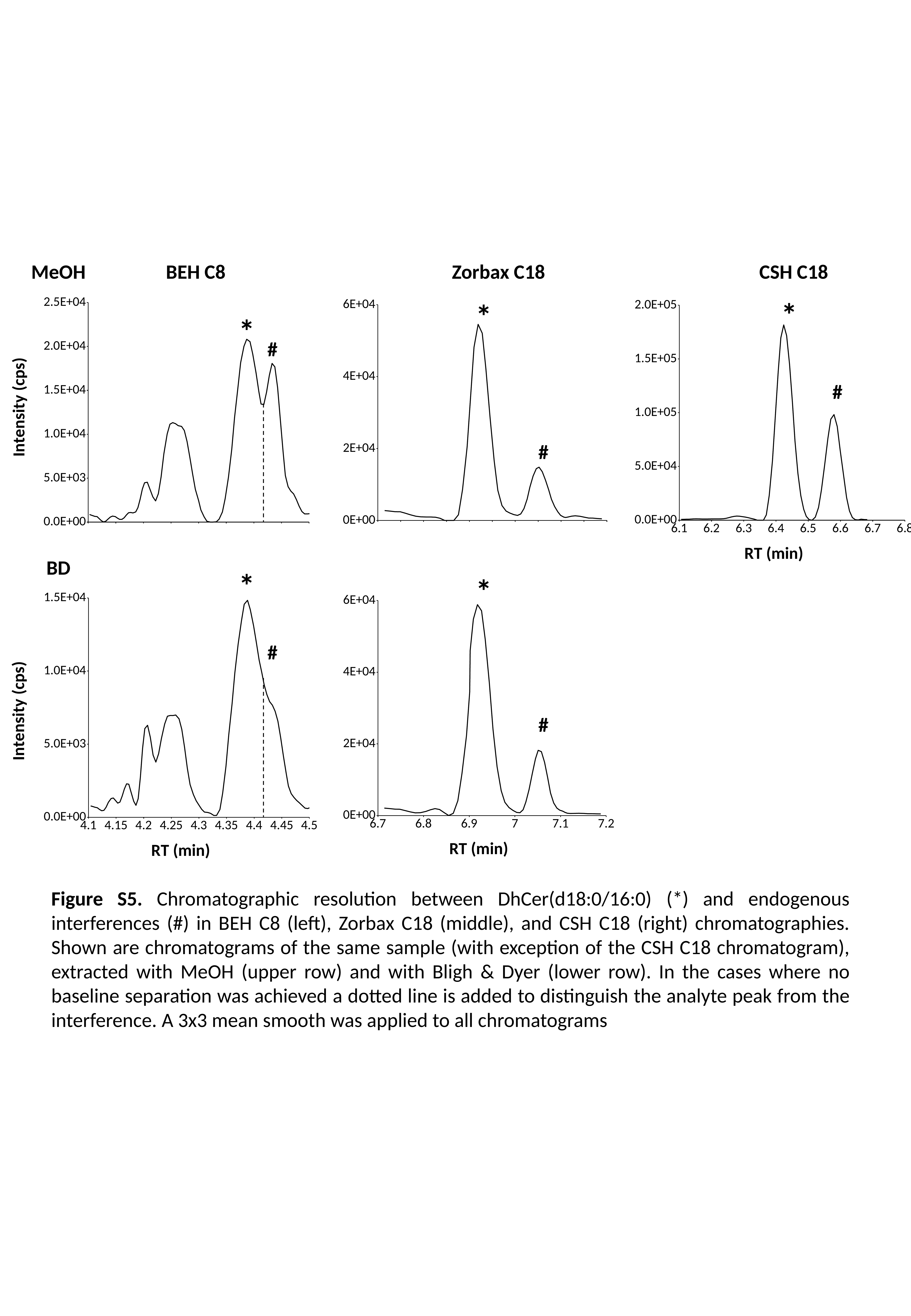

MeOH
BEH C8
Zorbax C18
CSH C18
*
### Chart
| Category | DhCer(18:1/16:0) |
|---|---|
### Chart
| Category | DhCer(18:1/16:0) |
|---|---|
### Chart
| Category | DhCer(18:1/16:0) |
|---|---|*
*
#
#
#
BD
*
*
### Chart
| Category | DhCer(18:1/16:0) |
|---|---|
### Chart
| Category | DhCer(18:1/16:0) |
|---|---|#
#
Figure S5. Chromatographic resolution between DhCer(d18:0/16:0) (*) and endogenous interferences (#) in BEH C8 (left), Zorbax C18 (middle), and CSH C18 (right) chromatographies. Shown are chromatograms of the same sample (with exception of the CSH C18 chromatogram), extracted with MeOH (upper row) and with Bligh & Dyer (lower row). In the cases where no baseline separation was achieved a dotted line is added to distinguish the analyte peak from the interference. A 3x3 mean smooth was applied to all chromatograms

## Slide 7
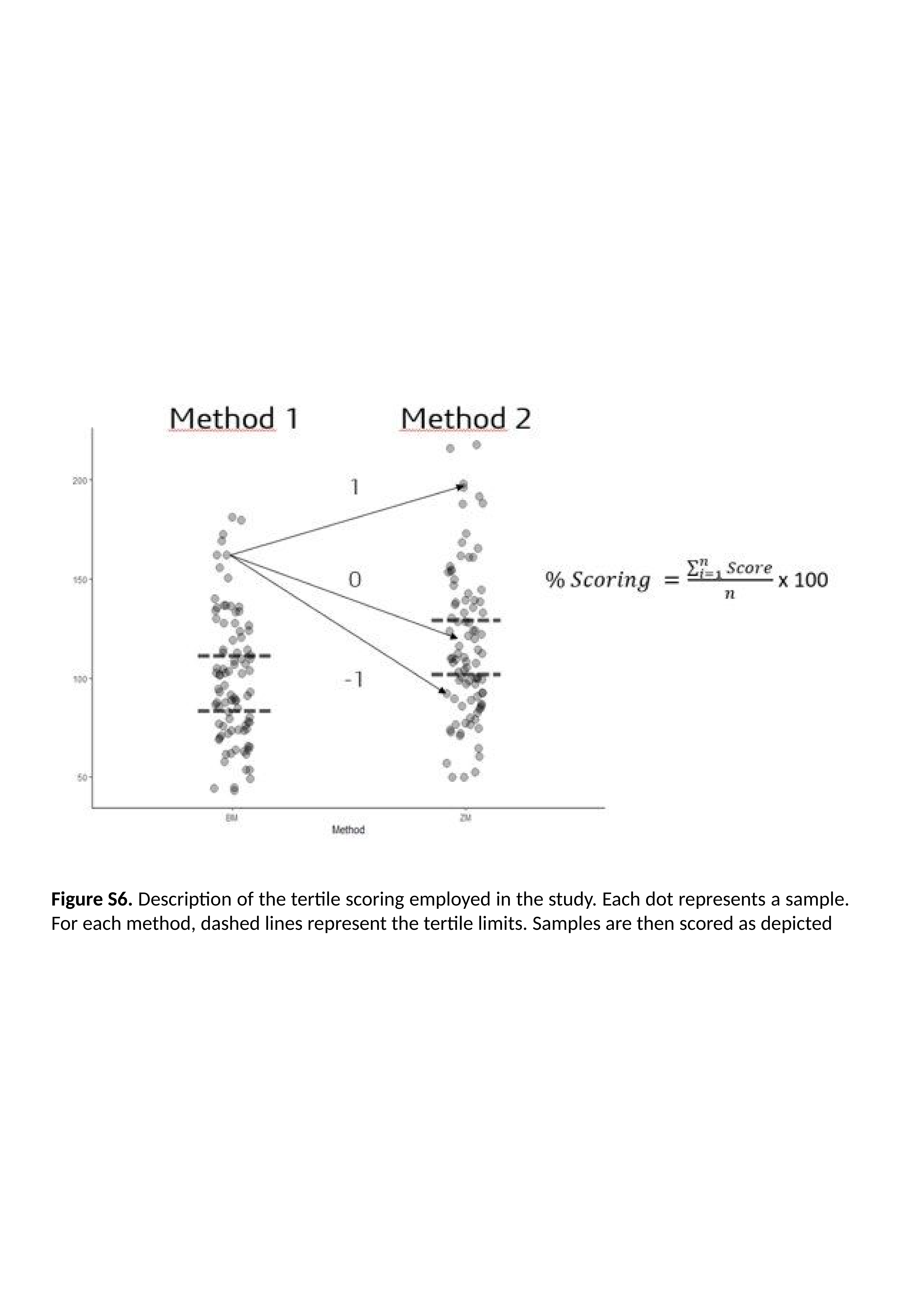

Figure S6. Description of the tertile scoring employed in the study. Each dot represents a sample. For each method, dashed lines represent the tertile limits. Samples are then scored as depicted

## Slide 8
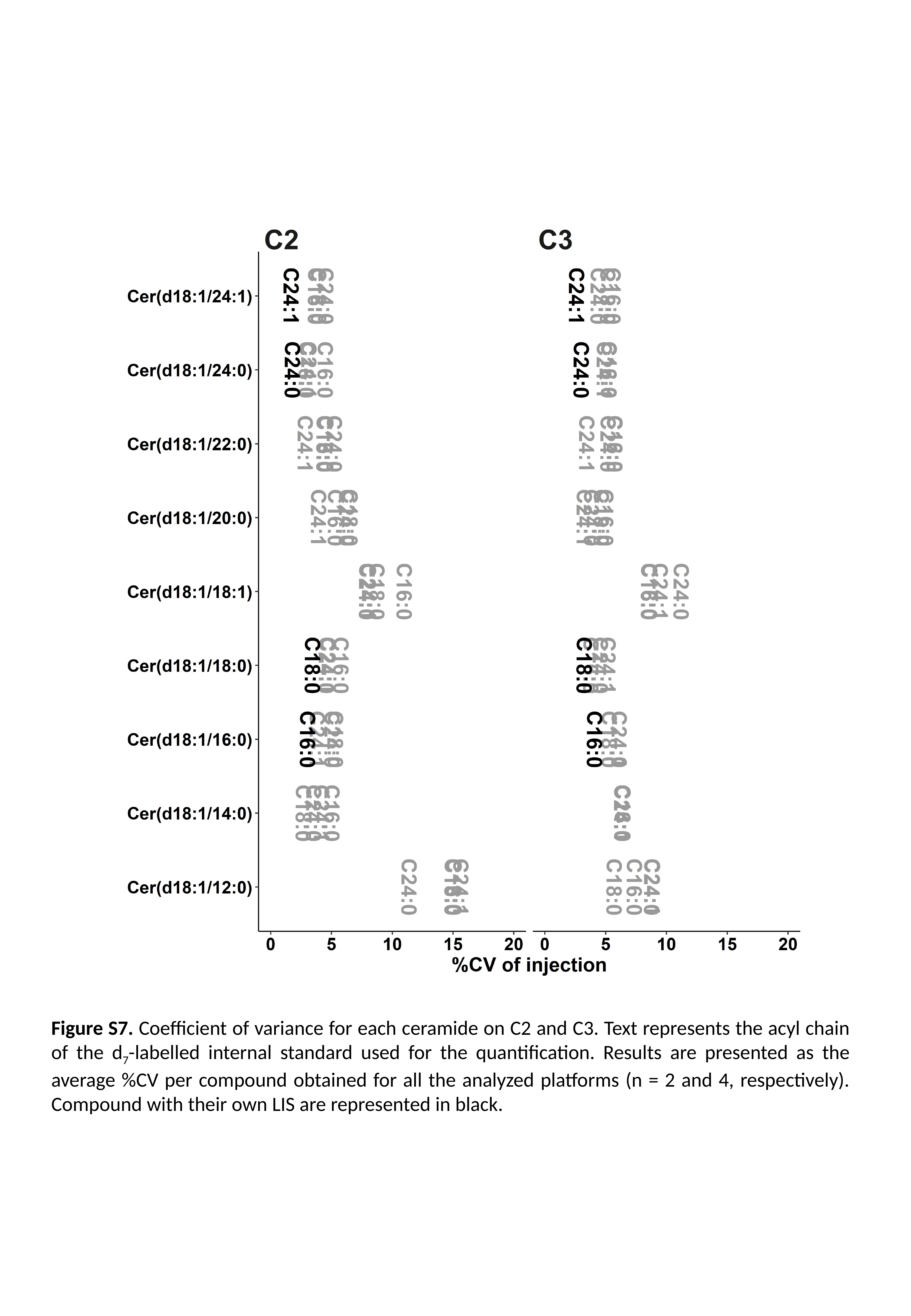

Figure S7. Coefficient of variance for each ceramide on C2 and C3. Text represents the acyl chain of the d7-labelled internal standard used for the quantification. Results are presented as the average %CV per compound obtained for all the analyzed platforms (n = 2 and 4, respectively). Compound with their own LIS are represented in black.

## Slide 9
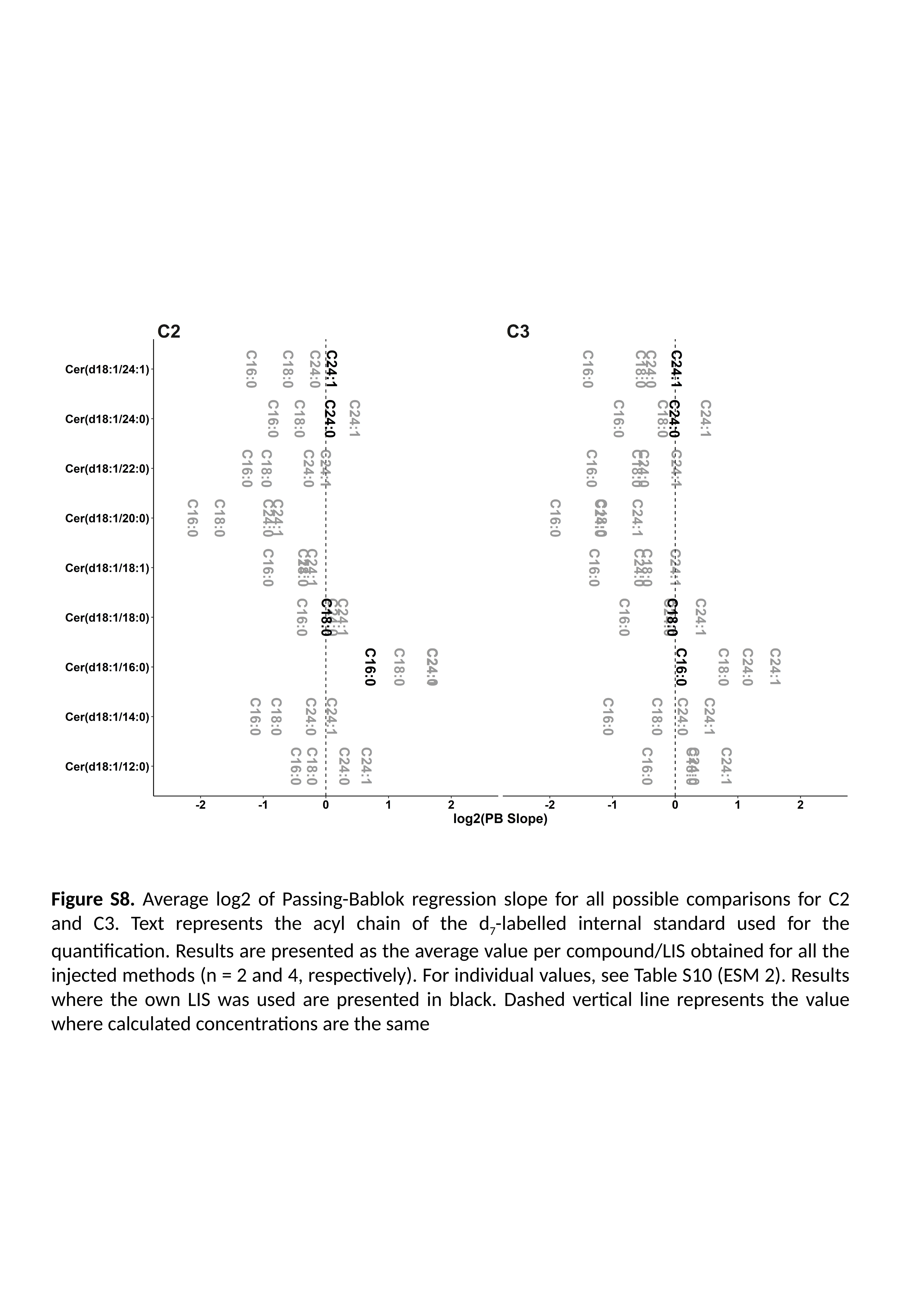

Figure S8. Average log2 of Passing-Bablok regression slope for all possible comparisons for C2 and C3. Text represents the acyl chain of the d7-labelled internal standard used for the quantification. Results are presented as the average value per compound/LIS obtained for all the injected methods (n = 2 and 4, respectively). For individual values, see Table S10 (ESM 2). Results where the own LIS was used are presented in black. Dashed vertical line represents the value where calculated concentrations are the same

## Slide 10
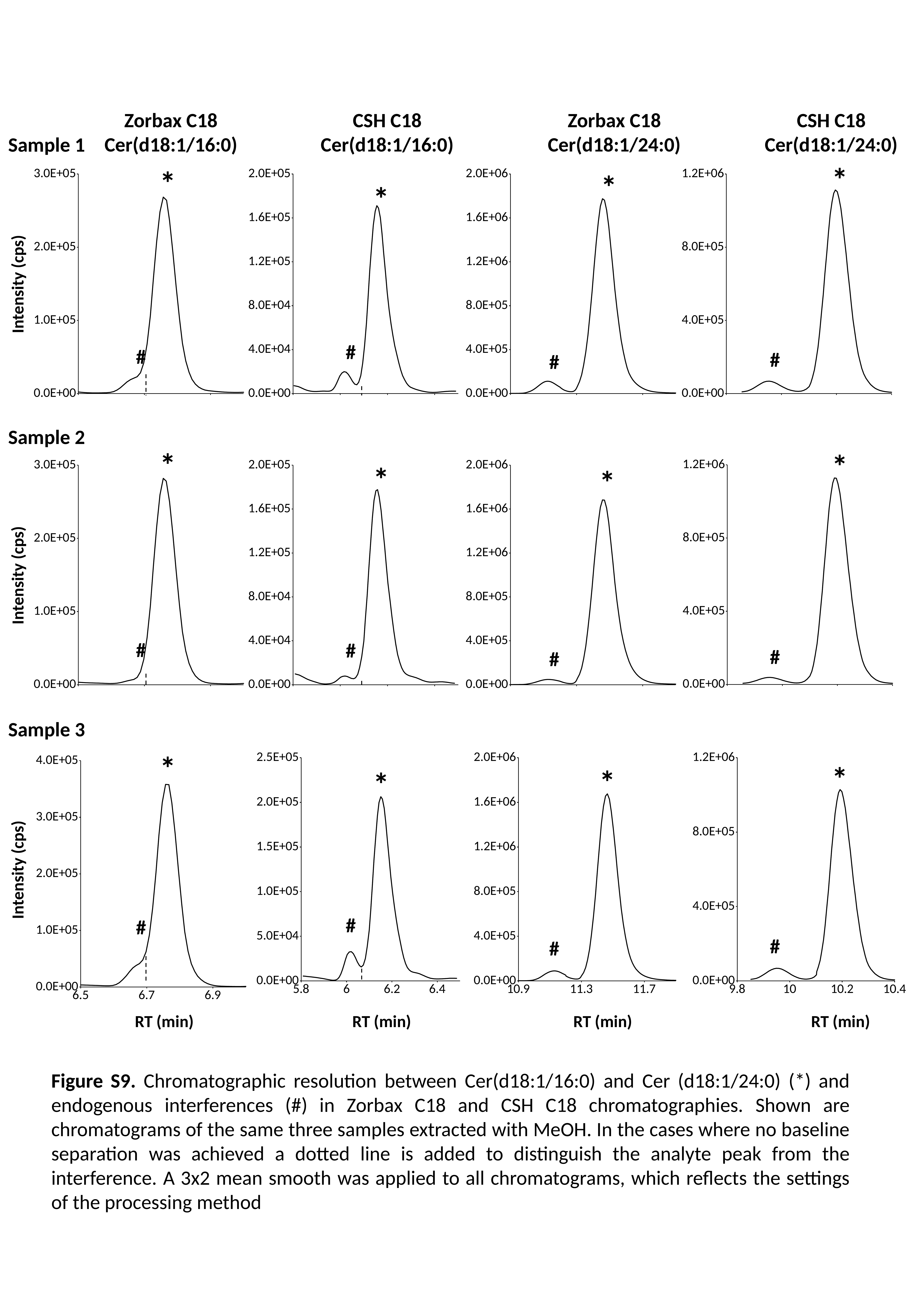

Zorbax C18
Cer(d18:1/16:0)
CSH C18
Cer(d18:1/16:0)
Zorbax C18
Cer(d18:1/24:0)
CSH C18
Cer(d18:1/24:0)
Sample 1
*
*
### Chart
| Category | |
|---|---|
### Chart
| Category | |
|---|---|
### Chart
| Category | |
|---|---|
### Chart
| Category | |
|---|---|*
*
Intensity (cps)
#
#
#
#
Sample 2
*
*
### Chart
| Category | |
|---|---|
### Chart
| Category | |
|---|---|
### Chart
| Category | |
|---|---|
### Chart
| Category | |
|---|---|*
*
Intensity (cps)
#
#
#
#
Sample 3
### Chart
| Category | |
|---|---|
### Chart
| Category | |
|---|---|
### Chart
| Category | |
|---|---|*
### Chart
| Category | |
|---|---|*
*
*
Intensity (cps)
#
#
#
#
RT (min)
RT (min)
RT (min)
RT (min)
Figure S9. Chromatographic resolution between Cer(d18:1/16:0) and Cer (d18:1/24:0) (*) and endogenous interferences (#) in Zorbax C18 and CSH C18 chromatographies. Shown are chromatograms of the same three samples extracted with MeOH. In the cases where no baseline separation was achieved a dotted line is added to distinguish the analyte peak from the interference. A 3x2 mean smooth was applied to all chromatograms, which reflects the settings of the processing method

## Slide 11
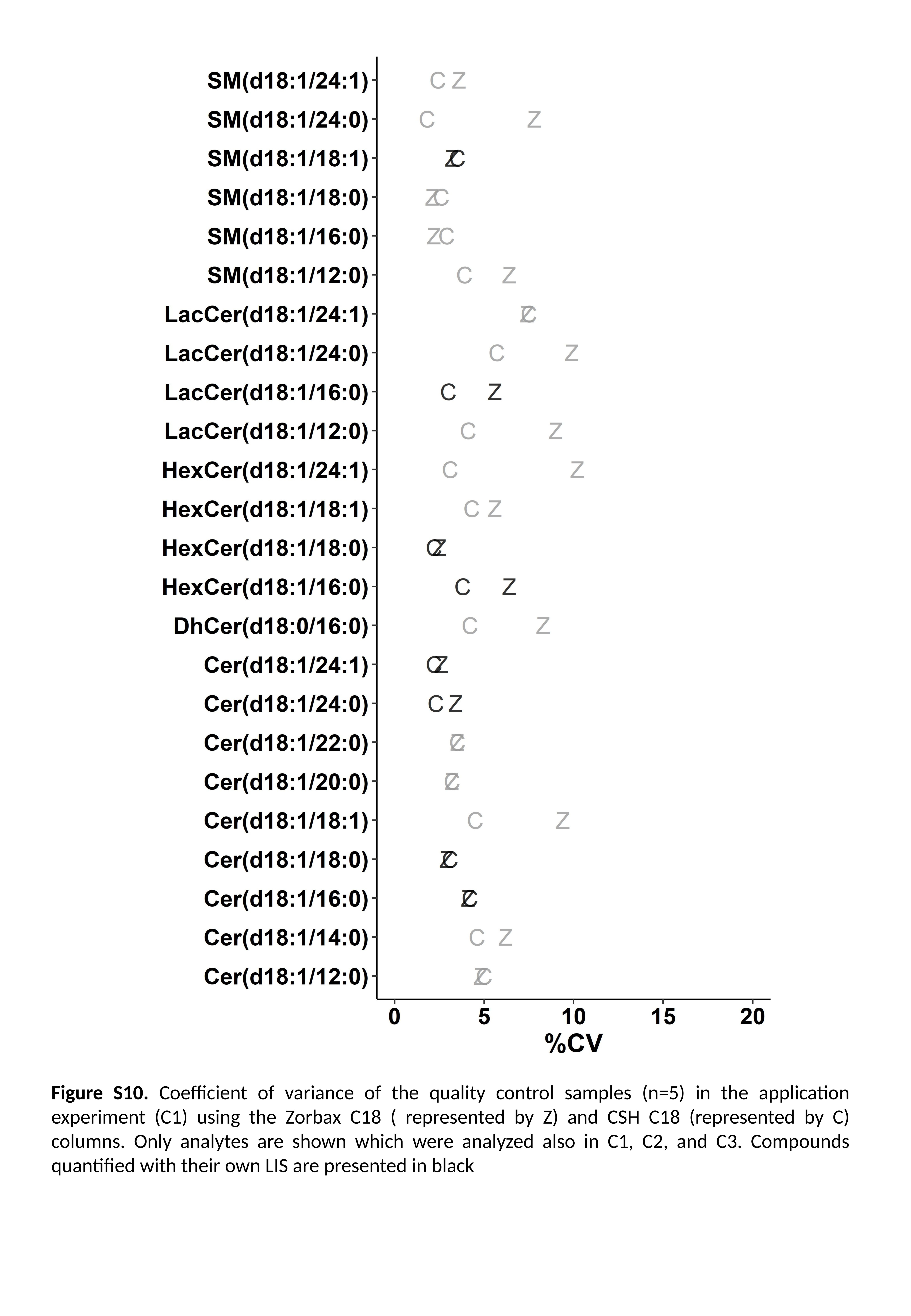

Figure S10. Coefficient of variance of the quality control samples (n=5) in the application experiment (C1) using the Zorbax C18 ( represented by Z) and CSH C18 (represented by C) columns. Only analytes are shown which were analyzed also in C1, C2, and C3. Compounds quantified with their own LIS are presented in black

## Slide 12
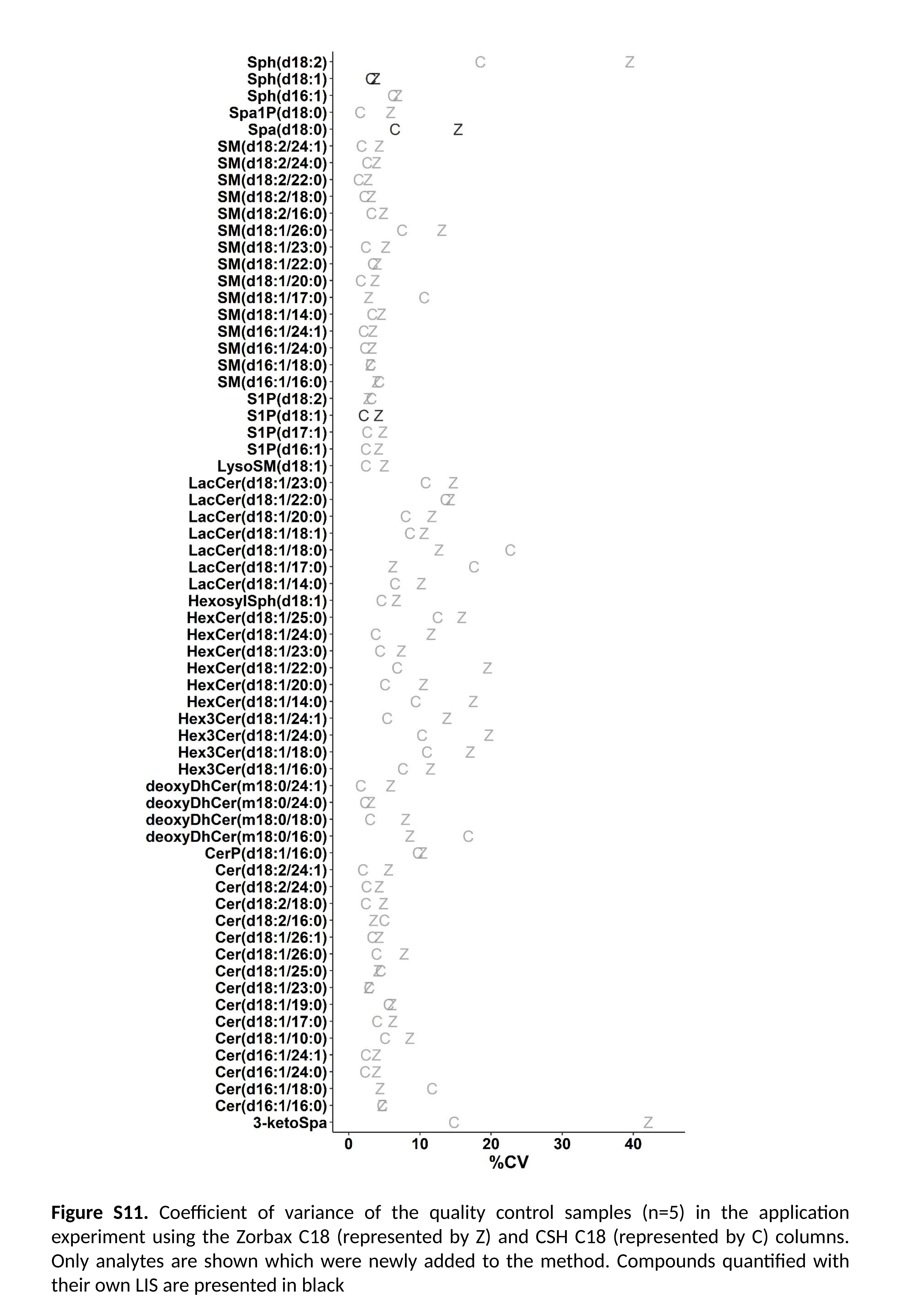

Figure S11. Coefficient of variance of the quality control samples (n=5) in the application experiment using the Zorbax C18 (represented by Z) and CSH C18 (represented by C) columns. Only analytes are shown which were newly added to the method. Compounds quantified with their own LIS are presented in black

## Slide 13
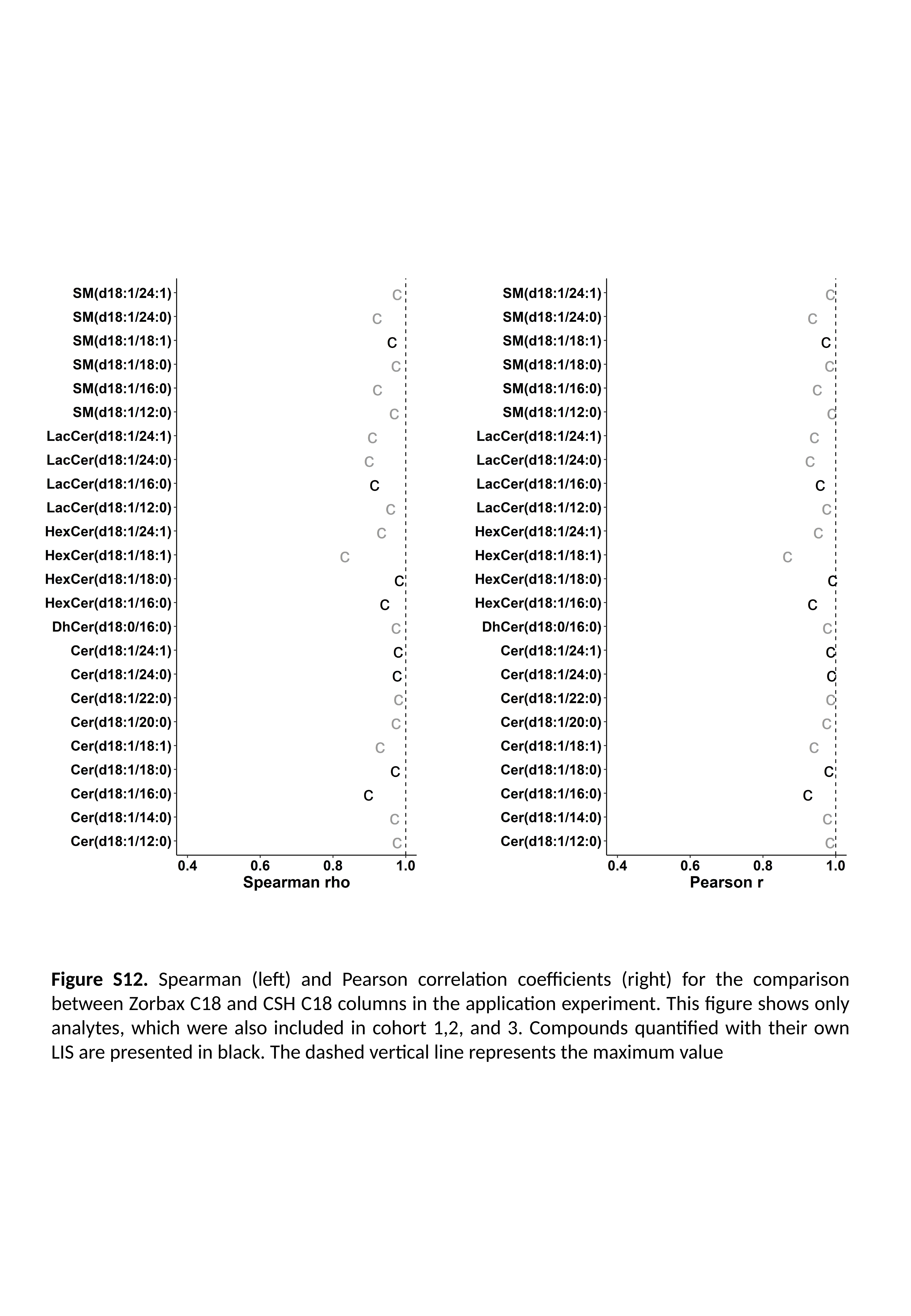

Figure S12. Spearman (left) and Pearson correlation coefficients (right) for the comparison between Zorbax C18 and CSH C18 columns in the application experiment. This figure shows only analytes, which were also included in cohort 1,2, and 3. Compounds quantified with their own LIS are presented in black. The dashed vertical line represents the maximum value

## Slide 14
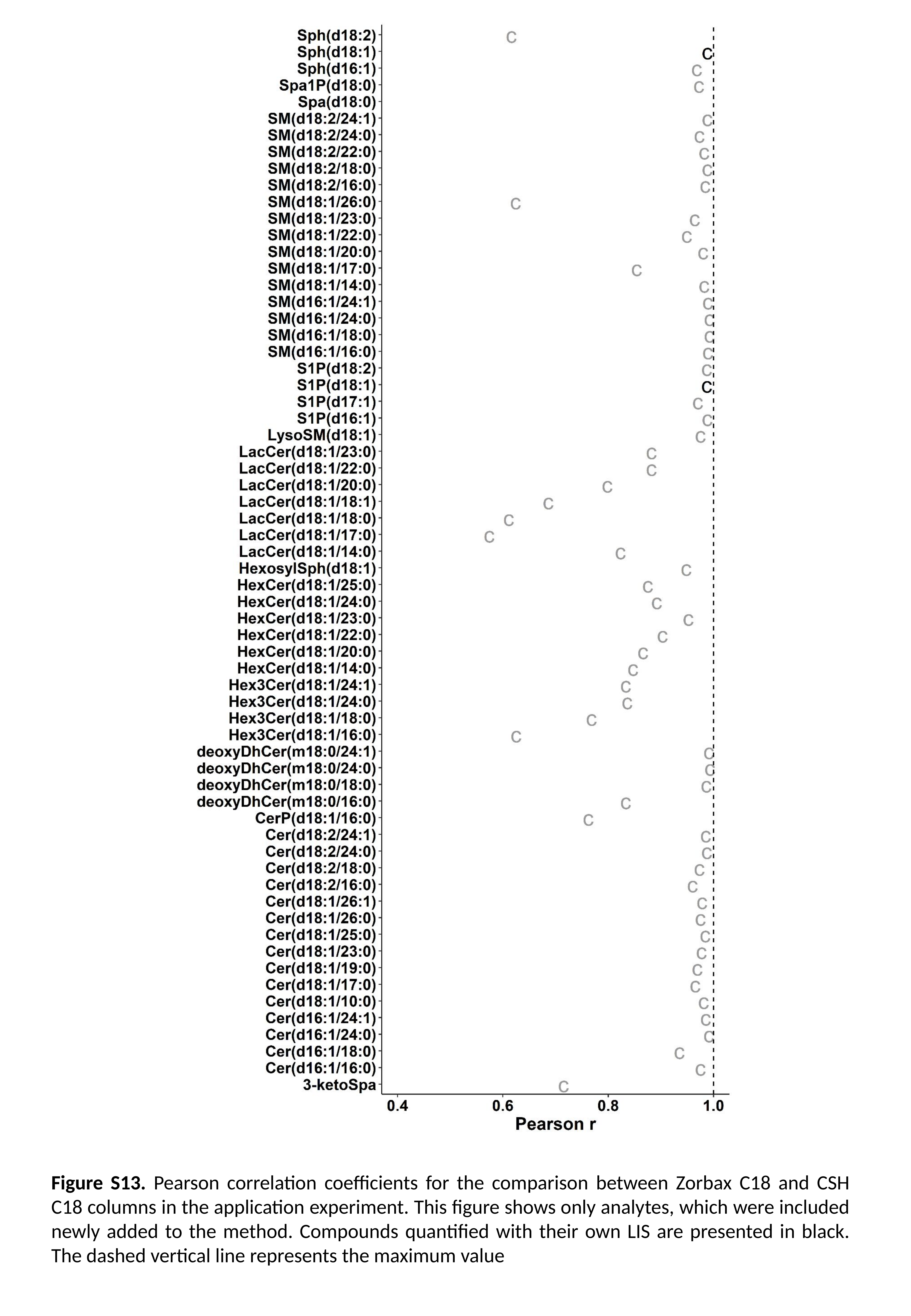

Figure S13. Pearson correlation coefficients for the comparison between Zorbax C18 and CSH C18 columns in the application experiment. This figure shows only analytes, which were included newly added to the method. Compounds quantified with their own LIS are presented in black. The dashed vertical line represents the maximum value

## Slide 15
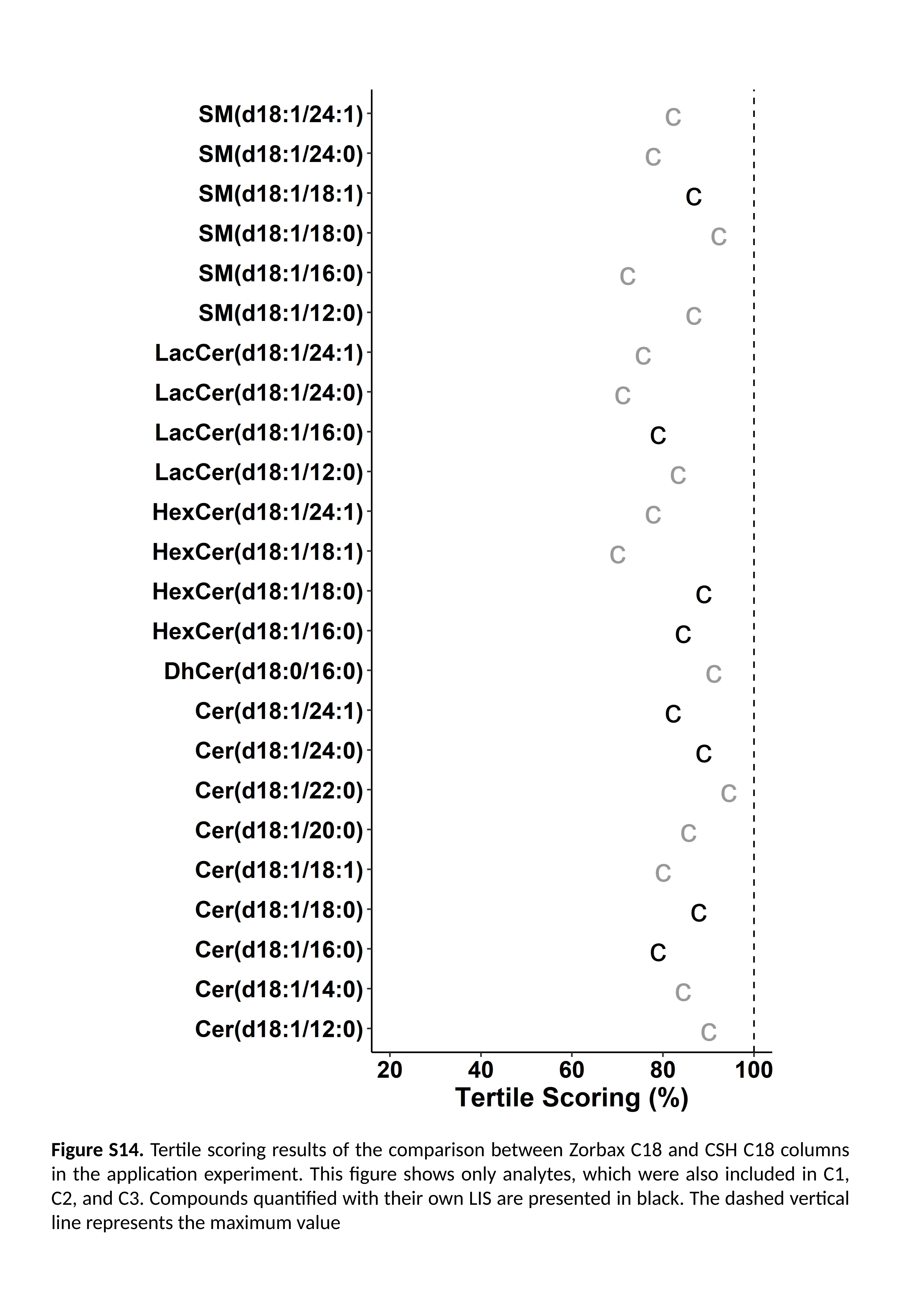

Figure S14. Tertile scoring results of the comparison between Zorbax C18 and CSH C18 columns in the application experiment. This figure shows only analytes, which were also included in C1, C2, and C3. Compounds quantified with their own LIS are presented in black. The dashed vertical line represents the maximum value

## Slide 16
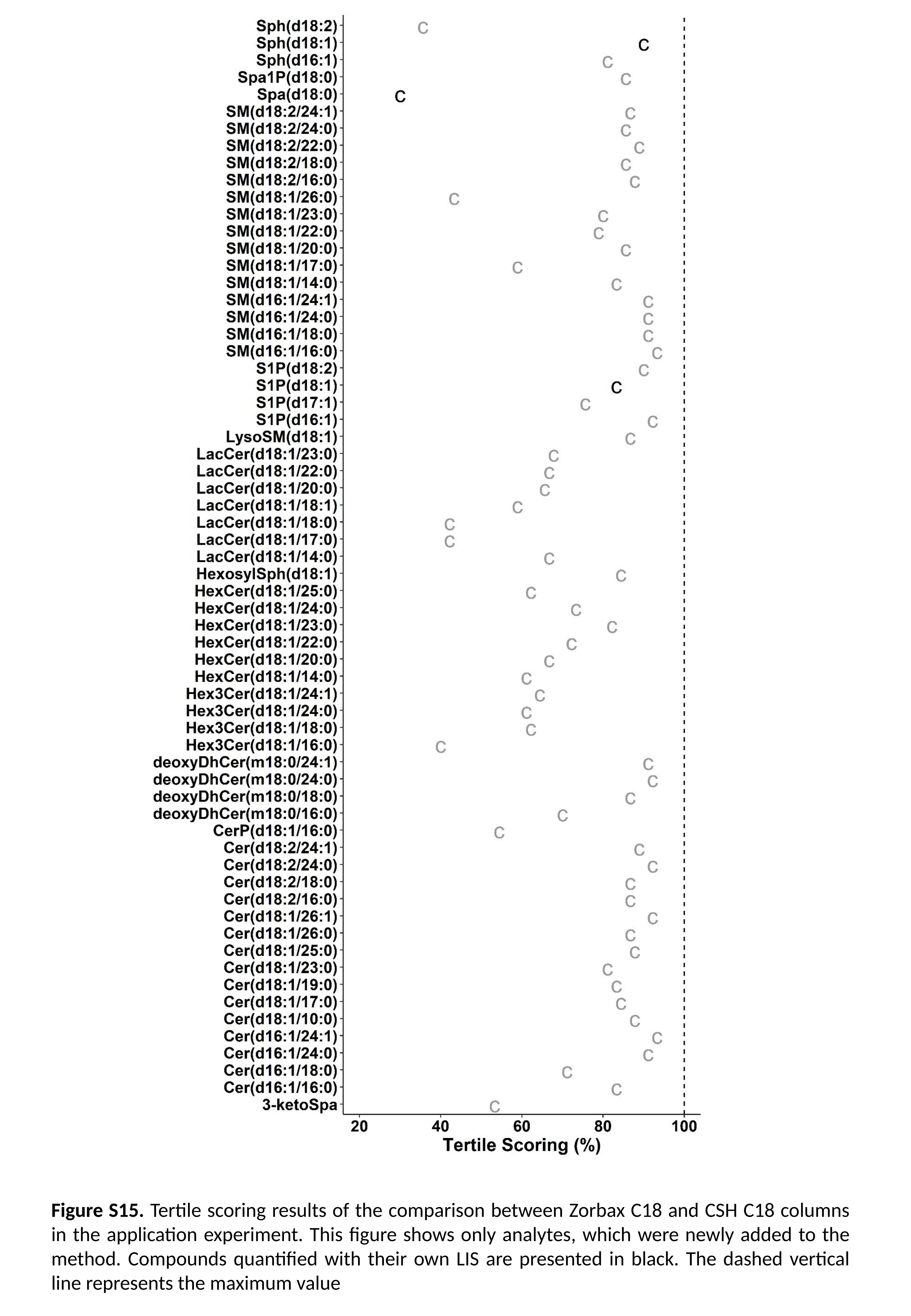

Figure S15. Tertile scoring results of the comparison between Zorbax C18 and CSH C18 columns in the application experiment. This figure shows only analytes, which were newly added to the method. Compounds quantified with their own LIS are presented in black. The dashed vertical line represents the maximum value

## Slide 17
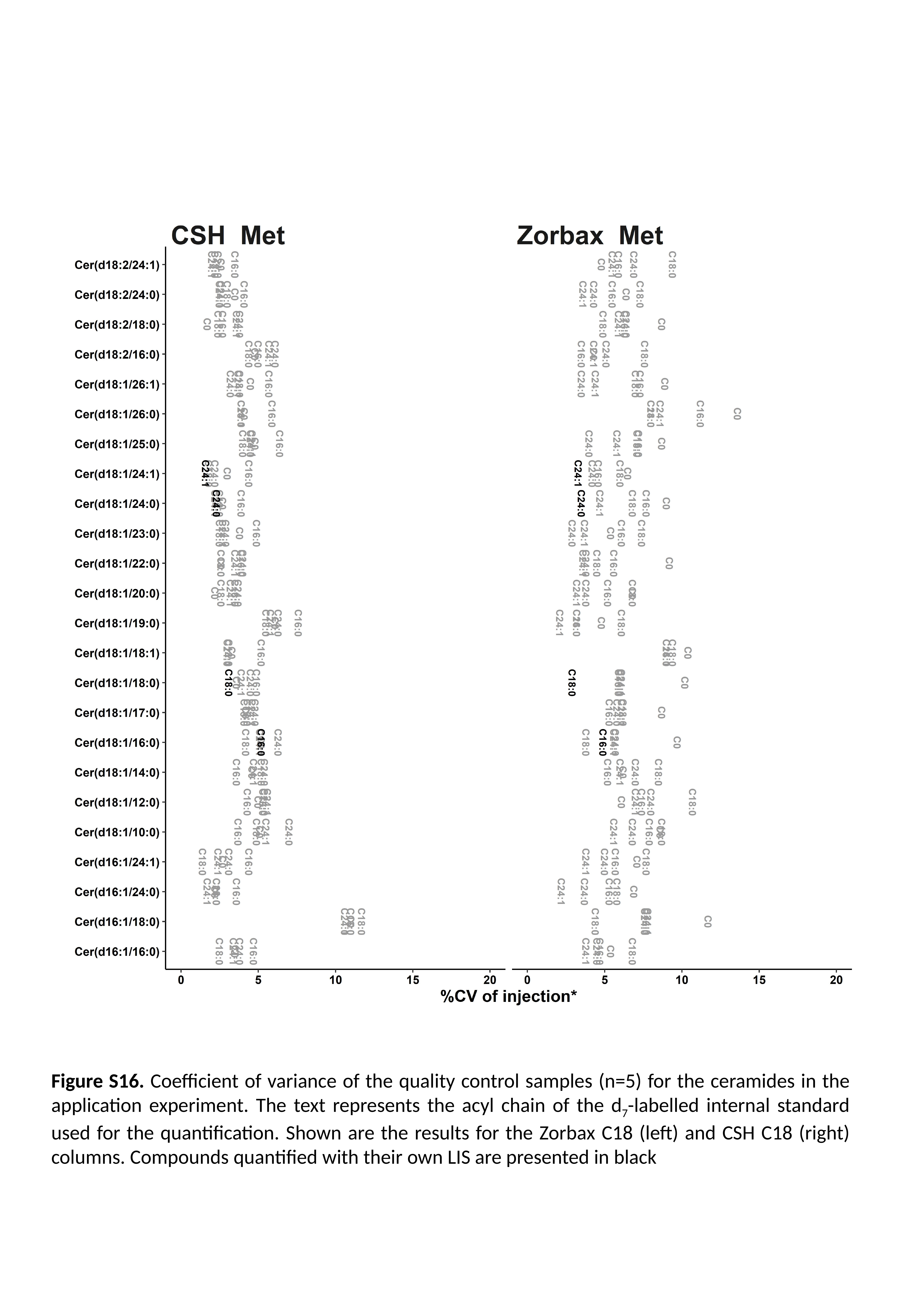

Figure S16. Coefficient of variance of the quality control samples (n=5) for the ceramides in the application experiment. The text represents the acyl chain of the d7-labelled internal standard used for the quantification. Shown are the results for the Zorbax C18 (left) and CSH C18 (right) columns. Compounds quantified with their own LIS are presented in black

## Slide 18
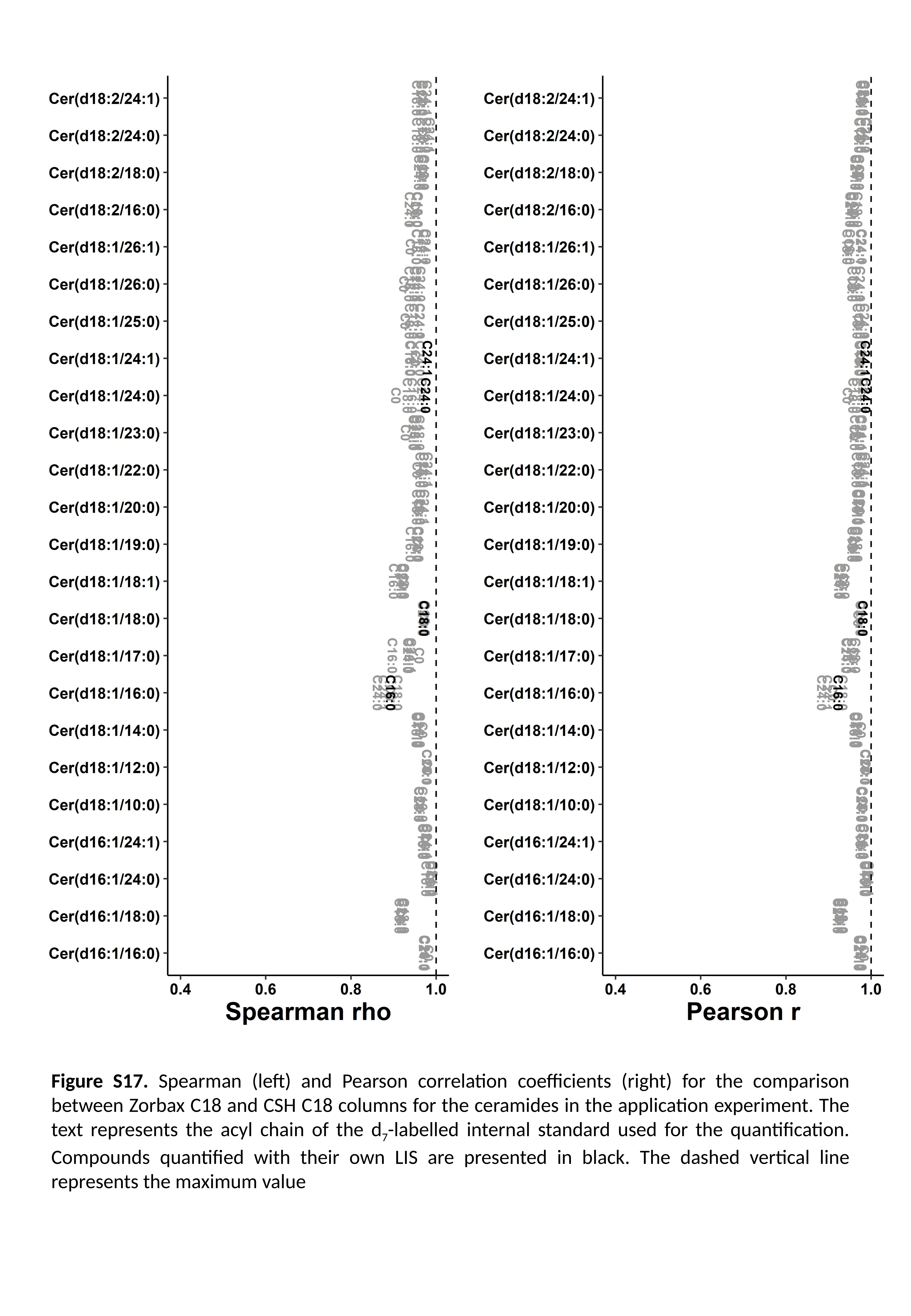

Figure S17. Spearman (left) and Pearson correlation coefficients (right) for the comparison between Zorbax C18 and CSH C18 columns for the ceramides in the application experiment. The text represents the acyl chain of the d7-labelled internal standard used for the quantification. Compounds quantified with their own LIS are presented in black. The dashed vertical line represents the maximum value

## Slide 19
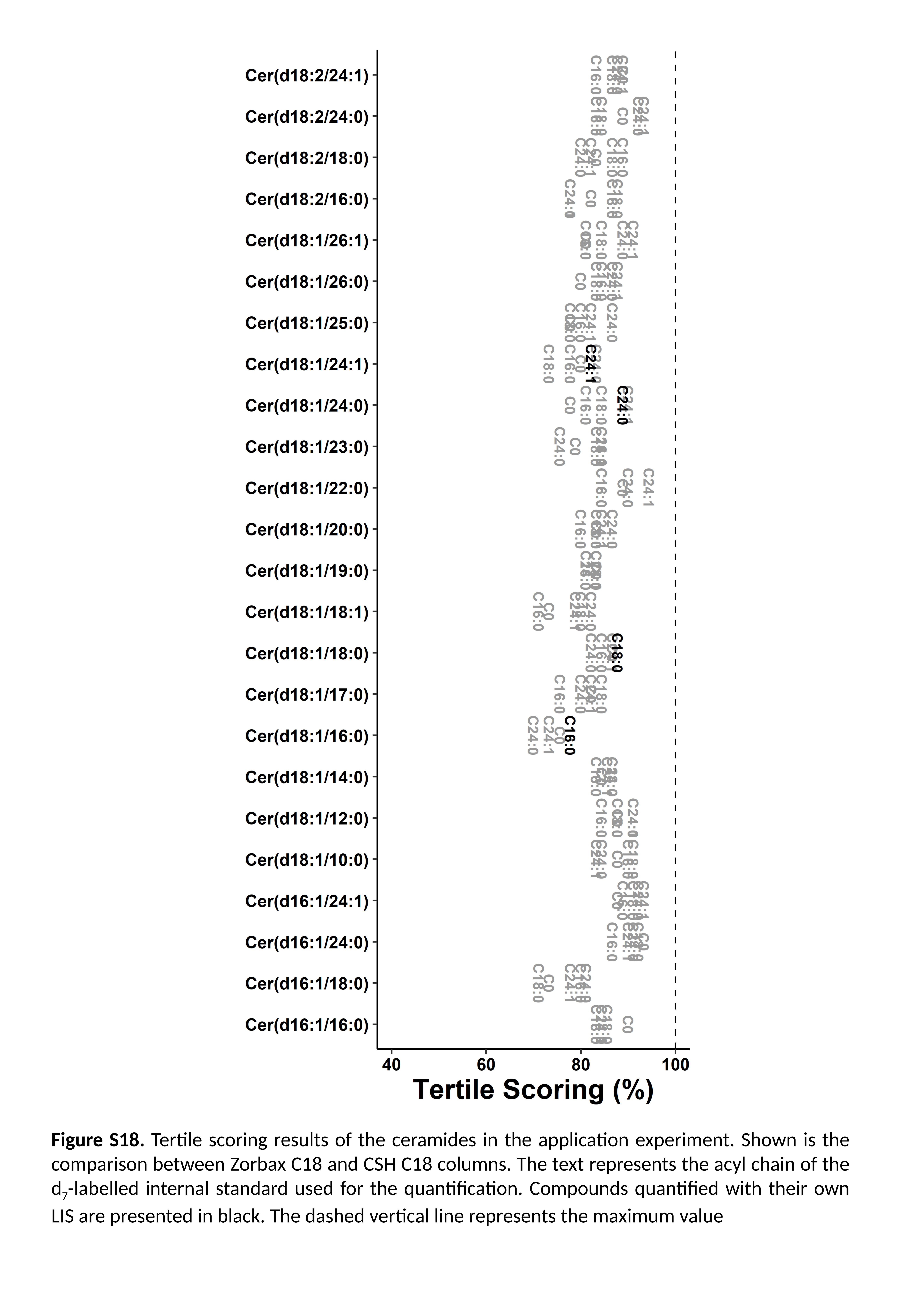

Figure S18. Tertile scoring results of the ceramides in the application experiment. Shown is the comparison between Zorbax C18 and CSH C18 columns. The text represents the acyl chain of the d7-labelled internal standard used for the quantification. Compounds quantified with their own LIS are presented in black. The dashed vertical line represents the maximum value
